# High Mitochondrial DNA Stability in B-Cell Chronic Lymphocytic Leukemia

**DOI:** 10.1371/journal.pone.0007902

**Published:** 2009-11-18

**Authors:** María Cerezo, Hans-Jürgen Bandelt, Idoia Martín-Guerrero, Maite Ardanaz, Ana Vega, Ángel Carracedo, África García-Orad, Antonio Salas

**Affiliations:** 1 Unidade de Xenética, Instituto de Medicina Legal, and Departamento de Anatomía Patolóxica y Ciencias Forenses, Facultade de Medicina, Universidade de Santiago de Compostela, Santiago de Compostela, Galicia, Spain; 2 Department of Mathematics, University of Hamburg, Hamburg, Germany; 3 Laboratorio Interdepartamental de Medicina Molecular, Departamento de Genética Antropología Física y Fisiología Animal, Facultad de Medicina, Universidad del País Vasco- Euskal Herriko Unibertsitatea, Leioa, Spain; 4 Servicio de Hematología, Hospital Txagorritxu, Vitoria, Spain; 5 Fundación Pública Galega de Medicina Xenómica (FPGMX), Hospital Clínico Universitario, Universidad de Santiago de Compostela, Galicia, Spain; Stanford University, United States of America

## Abstract

**Background:**

Chronic Lymphocytic Leukemia (CLL) leads to progressive accumulation of lymphocytes in the blood, bone marrow, and lymphatic tissues. Previous findings have suggested that the mtDNA could play an important role in CLL.

**Methodology/Principal Findings:**

The mitochondrial DNA (mtDNA) control-region was analyzed in lymphocyte cell DNA extracts and compared with their granulocyte counterpart extract of 146 patients suffering from B-Cell CLL; B-CLL (all recruited from the Basque country). Major efforts were undertaken to rule out methodological artefacts that would render a high false positive rate for mtDNA instabilities and thus lead to erroneous interpretation of sequence instabilities. Only twenty instabilities were finally confirmed, most of them affecting the homopolymeric stretch located in the second hypervariable segment (HVS-II) around position 310, which is well known to constitute an extreme mutational hotspot of length polymorphism, as these mutations are frequently observed in the general human population. A critical revision of the findings in previous studies indicates a lack of proper methodological standards, which eventually led to an overinterpretation of the role of the mtDNA in CLL tumorigenesis.

**Conclusions/Significance:**

Our results suggest that mtDNA instability is not the primary causal factor in B-CLL. A secondary role of mtDNA mutations cannot be fully ruled out under the hypothesis that the progressive accumulation of mtDNA instabilities could finally contribute to the tumoral process. Recommendations are given that would help to minimize erroneous interpretation of sequencing results in mtDNA studies in tumorigenesis.

## Introduction

Chronic Lymphocytic Leukemia (CLL) becomes manifest in progressive accumulation of lymphocytes in the blood, bone marrow, and lymphatic tissues [1]. B-CLL is the most frequent form of leukemia in Western countries and represents 30% of all leukemic cases [2]. Studies on ethnic distribution of CLL in the world have shown considerable variation [3], Thus for instance, according to the American Cancer Society (http://www.cancer.org), about 15,490 new cases of CLL will be diagnosed in the United States during 2009, and about 4,390 people will die of CLL in this country during this year. For patients with progressing CLL, treatment with conventional doses of chemotherapy is not curative; selected patients treated with allogeneic stem cell transplantation achieved prolonged disease-free survival [4], [5]. The median survival for all patients ranges from 8 to 12 years in older trials [6]. CLL occurs primarily in middle-aged and elderly adults, with increasing frequency in successive decades of life. The clinical course of this disease progresses from an indolent lymphocytosis without other evident disease to one of generalized lymphatic enlargement with concomitant pancytopenia. Complications of pancytopenia, including haemorrhage and infection, represent a major cause of death in these patients [7]. Prognostic factors that may help predict clinical outcome include cytogenetic subgroup, immunoglobulin mutational status, ZAP-70, and CD38 (see for instance [1]). Staging is useful in CLL to predict prognosis and also to stratify patients to achieve comparisons for interpreting specific treatment results. Anemia and thrombocytopenia are the major adverse prognostic variables. Although CLL has no standard staging system, the most common ones are the Rai staging system and the Binet classification [1], [8]. New prognostic markers have been proposed to the clinician and investigator [9].

The potential role of the mtDNA genome in CLL was first approached by Carew et al. [10] analyzing a sample of 20 patients. According to these authors, chemotherapy with DNA-damaging agents could cause mtDNA mutations in primary leukemia cells, which often exist in heteroplasmic condition. These findings were later put into question by Meierhofer et al. [11], who showed that platelet transfusion can mimic somatic mtDNA mutations. He et al. [12] sequenced the entire mtDNA from both normal tissue (buccal epithelial cells) and cells extracted from bone marrow in 24 patients with adult-onset leukemia. They reported mtDNA mutations in nine tumoral mtDNAs (one mutation per patient) and, in particular, inferred pathogenic implications of the mutation A15296G in leukemia. Grist et al. [13] analyzed mtDNA mutations in 22 patients with acute myeloid leukemia (AML) and 26 patients with acute lymphoblastic leukemia (ALL). The authors found (multiple) mtDNA control-region mutations in 36% of the AML patients and in 58% of the ALL patients, but the sequence information was not provided for closer examination. It was pointed out by the authors that most of the mutations tended to appear at hotspots: “*several hotspots were at sites of poly C tracts, but there was no single-sequence motif which seemed to be associated with mutations*” [13]. In a comprehensive review, Gattermann [14] analyzed the implications of mtDNA mutations in leukemogenesis. Recently, Yao et al. [15] studied mtDNA sequence variation in more than 3,500 single normal cells and individual blasts from 18 patients with leukemia and 10 healthy donors. Further they found that the somatic mutation process in leukemia is complex and generally leads to diverse levels of genetic alterations. These authors also observed that the somatic mutation events in single hematopoietic cells are prone to occur at well-characterized mutation hot spots, thus corroborating the results previously obtained in tumors of the central nervous system [16].

He et al. [12] conducted the first mtDNA study that aimed at comparing mtDNA extracted from leukemic cells with mtDNA extracted from buccal mucosa cells from the same patient, observing that 40% of their patients bear somatic point mutations in their mtDNAs. Other studies[17], [18] compared mtDNA from clonal bone marrow disorders with mtDNA obtained from healthy individuals; as advanced by Gattermann [14], these studies however may be futile since these comparisons just reflect the biological differences that exist between mtDNA lineages within or between human population groups (see also [19]). It has been claimed in numerous studies that the mtDNA molecule is in general prone to instability in tumorigenesis, and even hypothesized that mtDNA genome instability could play some active role during the development of the tumor in several types of cancer. Most of these studies, however, are most problematic in view of the patterns of recorded mtDNA alterations between putatively matched tissues, which are more akin to the result of sample mixing [19], [20], [21]. This prompted us to take a closer look at the previous mtDNA sequencing results in patients with CLL, AML, and ALL (especially those from Carew et al. [10]) in order to exhibit patterns that would clearly point to artefacts or other shortcomings.

To shed more light on the debatable issue of mtDNA alterations in leukemia we analyzed mtDNA instability in a large number of B-CLL patients from the same geographic area (Basque country) under strict laboratory conditions. Since previous studies always found alterations in the control-region, we sequenced the entire mtDNA control-region in lymphocytes obtained from blood samples of the B-CLL patients. Since the myeloid line and, in particular, the granulocytes should not be directly affected in B-CLL patients, these cells were used as the corresponding control samples for instability in the lymphoid line of each patient. In contrast to previous attempts, our design involved the comparison of two different cell lines that have very recent common cell ancestors (the colony deriving from the pluri-potent stem cells) in order to maximally rule out the effect of tissue-mediated instabilities. Such instabilities exist even when comparing different fragments of hairs from the same (healthy) individual [22], [23].

## Materials and Methods

### Ethical Statement

The study was conducted according to the Spanish Law for Biomedical Research (Law 14/2007- 3 of July) and complied with the Declaration of Helsinki. The study and the use of archive samples for this project was approved by the Ethics Committee of the University of Santiago de Compostela where the study was carried out. Written informed consent was obtained for all patients. All the samples were collected anonymously.

### Sample Collection and DNA Extraction

A total of 146 blood samples from patients diagnosed of B-CLL were collected (2–4 ml) by venous puncture using EDTA as anticoagulant. The patients belong to the sanitary area of the Hospital de Tagorritxu and Basurto in the Basque country. Written informed consent was obtained for all individuals.

Granulocyte and lymphocyte cells were separated using a gradient Ficoll-Paque Plus (Amhersham Biosciences) using manufacture protocols (see also Carew et al. [10] for a similar approach). According to manufacture instructions, we did not remove excess Ficoll-Paque PLUS in order to minimize contamination of the lymphocytes fraction with granulocytes. Platelets were removed as indicated in those instructions. These protocols minimize the presence of non-lymphocyte cells in the lymphocyte fraction and lead to the purification of the lymphoid extraction for the presence of lymphocytes.

We also recovered the granulocyte cell fraction. According to manufacture instructions, the efficiency of the separation is as follows: lymphocytes ∼95% of cells present in fraction are mononucleocytes, and ∼60% recovery of lymphocytes from the original blood sample. There are other cells in the extract: ∼3% granulocytes, ∼3.5% erythrocytes, and <0.5% of total platelets in the original blood sample. By using washing protocols, we intended to further improve the cell separation and purification of the lymphocyte and granulocyte cell fractions. Note also that standard sequencing protocols cannot detect the presence of a minor non-lymphoid cell component in the lymphocyte extract if this component is below ∼10% of the mixture. In any case, the presence of such cell mixture would not mask the mutational difference (if any) between both cell lines; this difference would be generally detected as a heteroplasmic-like pattern.

After the two fractions were obtained, we extracted the DNA using a standard phenol-chloroform protocol [24]; quantification was performed using GeneQuant Pro (Pharmacia), which showed variable concentrations varying between 10 to 100 ng/µl.

### PCR Amplification and Automatic Sequencing of the mtDNA Control-Region

The PCR was performed in 22 µl of reaction mixture containing 50 µM of each dNTP (200 µM of GeneAmp® 10 mM dNTP Mix with dTTP, Applied Biosystems [AB], Foster City, CA, USA), 2,5U *Taq* DNA Polymerase, recombinant (Invitrogen, Carlsbad, CA, USA), 1X PCR Buffer, 1,5 mM of Magnesium Chloride, 016 µM of BSA, 0,2 µM of each primer forward and reverse (15997L and 017H for HVS-I, and 16555L and 599H for HVS-II; Table 1), water-up to 22 µl and 3 µl sample template. PCR amplification was carried out in a thermocycler GenAmp PCR System 9700 (AB) with the following conditions: one cycle of 95°C for 1 minute; then 35 cycles of 95°C for 10 seconds, 55°C for 30 seconds and 72°C for 30 seconds, and ending 15°C for 10 minutes. For those samples showing insertions at 573, we additionally used the amplification pair of primer 332L [15] and primer 901H [25] in order to obtain forward and reverse reading (Table 1).

**Table 1:** Set the primers used for PCR amplification and sequencing.

mtDNA segment	PCR primer (5′ to 3′)		Sequencing primers (5′ to 3′)	
HVS-I (16024-16569)	15997L	CACCATTAGCACCCAAAGCT	15997L	CACCATTAGCACCCAAAGCT
	–	–	16254L	CACATCAACTGCAACTCCAAA
	–	–	16236H	CTTTGGAGTTGCAGTTGATG
	–	–	16401H	TGATTTCACGGAGGATGGTG
	–	–	16450H	CAAGTGTTATGGGCCCGGAGC
	–	–	16380L	TCAGATAGGGGTCCCTTGAC
	017H	CCCGTGAGTGGTTAATAGGGT	017H	CCCGTGAGTGGTTAATAGGGT
HVS-II (1-578)	16555L	CCCACACGTTCCCCTTAAAT	16555L	CCCACACGTTCCCCTTAAAT
	–	–	172L	ATTATTTATCGCACCTACGT
	–	–	285H	GGGGTTTGGTGGAAATTTTTTG
	332L	CCCGCTTCTGGCCACAGCAC	332L	CCCGCTTCTGGCCACAGCAC
	–	–	370L	CCCTAACACCAGCCTAACCA
	–	–	408H	CTGTTAAAAGTGCATACCGCCA
	599H	TTGAGGAGGTAAGCTACATA	599H	TTGAGGAGGTAAGCTACATA
	901H	ACTTGGGTTAATCGTGTGACC	901H	ACTTGGGTTAATCGTGTGACC

After this reaction, PCR products and negative controls were checked in polyacrylamide gel and visualized with silver staining. Then PCR products were purified to remove excess of primers and un-incorporated dNTPs in MultiScreen®PCRμ_96_ plates (Millipore, Bedford, MA, USA) according to the manufacturer protocol.

Sequencing reaction was performed in 11.5 µl of reaction mixture, containing 2.5 µl of sequencing buffer (5X), 0.5 µl of BigDye Terminator v3.1 Cycle Sequencing Kit (AB), 1 µl of the corresponding primer (final concentration was 1 µM), 3 µl of the purified PCR product and water up to 11.5 µl. Sequencing reaction was carried out in a thermocycler GenAmp PCR System 9700 (AB) with one cycle of 96°C for 3 minutes and then 25 cycles of 96°C for 30 seconds, 50°C for 15 seconds and 60°C for 4 minutes or was carried out in a 9800 Fast Thermal Cycler (AB) with one cycle of 96°C for 1 minute then 25 cycles of 96°C for 10 seconds, 50°C for 5 seconds and 60°C for 1 minute. To obtain ‘clean’ electropherograms, the sequencing product was doubly purified, first using Montage™ SEQ_96_ Sequencing Reaction Cleanup Kit (Millipore) according to manufacturer protocols, followed by purification with Sephadex™ G-10 (Amersham Biosciences, Uppsala, Sweden), the latter also according to manufacturer protocol. MtDNA automatic sequencing was carried out in a capillary electrophoresis ABI3730 (AB). Each pair of samples showing differences between granulocytes and lymphocytes were sequenced in both forward and reverse directions. In order to obtain clear pattern of instability or simply to allow the reading of length variability, additional *ad hoc* primers were used (the full list of primers is given in Table 1).

All instabilities found at this stage were first rechecked in the same laboratory through all the steps involving PCR amplification and sequencing in forward and reverse direction for both the granulocytes and the lymphocytes counterparts by using a slightly different protocol which allows to minimize potential technical artefacts. The PCR was performed in 10 µl of reaction mix, containing 4 µl of *Taq* PCR Master Mix (Qiagen, Hilden, Germany), 0.5 µl 1 µM of each primer, 1 µl sample template and 4 µl of water. This PCR was carried out in a thermocycler GenAmp PCR System 9700 (AB) with one cycle of 95°C for 15 minutes and then 35 cycles of 94°C for 30 seconds, 58°C for 90 seconds and 72°C for 90 seconds with a full extension cycle of 72°C for 10 minutes. The PCR product was purified with ExoSAP-IT (Amersham Biosciences): 2.15 µl of PCR product was incubated with 0.85 µl ExoSAP-IT for 20 min at 37°C followed by 15 min at 80°C for enzyme inactivation. The next steps (e.g. sequencing, purification) were carried out using the protocol described above.

### Assessing Sequence Quality

The SeqScape v.2.5 (AB) was set up to automatically detect the presence of heteroplasmic-like patterns involving at least 15% of the signal for the minor variant; in addition, all the electropherograms were inspected visually. In order to avoid erroneous interpretation of seeming DNA instabilities [19] we have followed a phylogenetic framework [19], [26], [27], [28] that allowed us to detect some errors committed during the analytical and documentation process. Indeed, the analysis of DNA (usually through automatic sequencing) has always been prone to errors of different nature [26], [27], [29], [30], [31], [32], [33], [34], [35].

All sequence instabilities detected in our set of samples were finally confirmed by sequencing the forward and reverse strains and replicated in a different laboratory by a different analyst. The laboratory where the replication was carried out was not informed about the sequence profiles expected for the DNA samples in order to rule out any bias in reading and interpreting sequencing electropherograms. Control samples (those without any apparent instability) were also submitted to the second lab as well as samples showing seeming instabilities in the forward but not replicated in the reverse sequencing. All the probable instabilities observed in the first laboratory were confirmed in the second laboratory. The sequencing results obtained for all the samples are presented in Data S1.

### Identification of the Same Donor for Each Pair of Lymphocyte and Granulocyte Samples

For all those samples showing instability-like patterns we genotyped a set of microsatellites in the lymphocyte fraction and their counterpart granulocytes. This (along with sharing the same mtDNA profile) allowed us to corroborate a common biological source for these pairs of samples and therefore practically rule out potential sample mix-up. We have followed the protocols of the Instituto de Medicina Legal of the Universidad de Santiago de Compostela. The following STR autosomal markers were analyzed using PowerPlex® 16 System (Promega; Madison, USA): D21S11, D3S1358, PENTA-E, D16S539, CSF1PO, FGA, PENTA-D, TPOX, TH01, vWA, D8S1179, D18S51, D5S818, D7S820, D13S317, and amelogenin. Samples were additionally genotyping using PowerPlex® which contains the same STRs as PowerPlex® (with the exception of PENTA-D and PENTA-E) but has in addition the STR markers D19S433 and D2S1338.

### Phylogenetic Analysis and Database Comparisons

Polymorphisms are referred with respect to the revised Cambridge Reference Sequence (rCRS [36]). Haplogroup classification was carried out *alter alia* according to ref. [37], [38], [39], [40], [41], [42], [43], [44], and using the most up to date mtDNA tree from http://www.phylotree.org/. A worldwide mtDNA database of control-region sequences published in the literature and/or Genbank was used for searching mtDNA profiles; this database also contains Basque and other Iberian profiles (e.g. [45], [46], [47], [48]).

### Statistical Analysis

Pearson's Chi-squared test with Yates' continuity correction was computed in order to test for possible association between clinical-pathological variants (Table 2) and the amount of instabilities found and haplogroup status. In order to adjust *P*-values for multiple tests, we applied Bonferroni correction and the procedure [49] to control the False Discovery Rate at the level of *α* = 0.05: (i) for *m* tests, the *P*-values are ranked in ascending order *P*_(1)_≤*P*_(2)_≤…≤*P*_(m)_, (ii) denote by *H_(i)_* the null hypothesis corresponding to *P*_(i)_; let *k* be the largest *i* for which
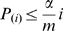
and (iii) all null hypotheses H_(1)_ … H_(k)_ are rejected.

**Table 2:** Clinico-pathological characteristics of patients with B-CLL.

Age (years)		
	range	33 to 92
	mean	69
**Gender**		
	Male	∼57%
	Female	∼43%
**Origin**		
	Araba (Basque Country, Spain)	∼90%
	Gupuzkoa (Basque Country; Spain)	∼1%
	Burgos (Castilla; North-central Spain)	∼1%
**Transplant**		
	none	100%
**Inmuno-phenotype**		
	19+ and 5+	∼95%
	FMC7-	∼95%
	79b-	∼92%
	23+	∼91%
	38-	∼85%
	10-	∼76%
	22-	∼66%
	k+	∼44%
	k-	∼33%
	a-	∼29%
	a+	∼26%
	22+	∼25%
	38+	∼8%
	79b+	∼4%
**CD38**		
	>30%	∼6%
	<30%	∼94%
**Serological markers**		
	LDH high	∼14%
	LDH normal	∼86%
	B2MG high	∼45%
	B2MG normal	∼55%
**Tissue morphology**		
	typical	∼82%
	atypical	∼18%
**Survival**		
	alive	∼92%
	exitus	∼8%
**RAI classification**		
	stage 0	∼54%
	stage I	∼31%
	stage II	∼6%
	stage III	∼1%
	stage IV	∼8%
**Binet staging**		
	stage A	∼88%
	stage B	∼2%
	stage C	∼10%
**Adenopaties**		
	yes	∼32%
	no	∼68%
**Marrow infiltration**		
	non-diffuse	∼87%
	diffuse	∼13%
**Electromagnetic radiation**		
	yes	∼7%
	no	∼83%
**Treatment**		
	none	∼68%
	Leukeran	∼18%
	Ciclosfosfamida	∼9%
	Fluradabina	∼8%
	Chop	∼8%
	Clorambucil	∼6%
	anti CD20	∼6%
	Prednisona	∼4%

Statistical evaluation of the common biological source for pairs of tumor/non-tumor samples showing mtDNA instabilities has been carried out according to standard forensic conventions for sample identification. Thus, the software Familias 1.81 [50] was used to compute the likelihood ratio (LR) that considers the probability of the evidence given two alternative hypotheses: the samples came from the same donor *versus* the samples came from different donors). LR was in all the cases higher than 10^13^ (Data S2).

## Results

### Reassessment of the Data from Carew et al. (2003)

Carew et al. [10] analyzed four mtDNA fragments from peripheral blood samples from 20 patients with B-CLL (10 untreated and 10 treated with chemotherapy). According to the authors, their results “*revealed that primary CLL cells from patients with prior chemotherapy has a significantly higher frequency of heteroplasmic mutations than did those from untreated patients*” [10]. Note however, that no attempt was made to determine the effect of chemotherapy in one and the same patient, and no attempt was made to distinguish mutations in the blood fraction (lymphocytes, in particular) from germline mutations. Therefore, from the outset, the design of the experiments is not really appropriate for understanding the role of mtDNA mutations in leukemia since there is no way to know whether the differences observed between treated *versus* untreated patients were due to normal variation between individuals rather than a higher rate of instability in treated patients.

The mtDNA regions that could be analyzed for mutations are the amplified fragments minus primer locations, i.e. 35–464 (covering the second hypervariable segment, HVS-II), 3324–3806, 7665–8296, 8560–9039, 11424–11905, and 15281–15752. The obtained sequence fragments were apparently compared to some version of the Cambridge Reference Sequence (CRS) but obviously not to the sequence NC_18007.4 as asserted by Carew et al. (2003), which e.g. bears the changes A73G, C150T, T195C, A263G, 309+C, 315+C, and T408A relative to the rCRS. The rCRS is a member of haplogroup H2a2, the root of which is distinguished from rCRS by the four changes A263G, 315+C, A8860G, and A15326G [51]. These changes should have been observed in almost all mtDNA sequences under study. However, the latter two mutations were nowhere recorded in those tables [10] and, in addition, the former two are lacking in patients UT7 and UT8.

The authors seem to have then interpreted their sequencing results under the wrong premise that the amount of differences to the rCRS could be indicative of cancer [19]. However, the worldwide phylogeny clearly shows that the rCRS is just one particular mtDNA lineage typically differing from other lineages by dozens of changes. The obtained sequencing results can be compared, one by one, to the entire database of complete mtDNA sequences. Leaving heteroplasmic changes aside for the moment, all 20 sequences except one testify to lineages of West Eurasian (and European, in particular) ancestry.

The mtDNA lineages of patients T1, T4, T5, T9, T10, UT1, UT2, and UT5 may all belong to haplogroup H (or at least to the larger haplogroup HV). In particular, T1, T5, and T9 bear the mutation C456T characteristic of haplogroup H5. Moreover, the three changes T195C, A257G (which is a quite rare event), and 309+C found in patient UT1 are shared with a particular lineage from haplogroup H1 (GenBank accession number EF177411 [52]). Patients T2 and T3 bear mtDNA lineages belonging to a specific branch of haplogroup K1a1a that have C114T; evidently, A73G and A263G were overlooked in Patient T3. Patients UT7 and UT8 have haplogroup K1a4a1 lineages, for which A263G and 315+C were not recorded; the nucleotide information for UT7 in column “Change” of the table is mis-documented. Patients T6, T7, and T10 have haplogroup U lineages, which cannot be further specified except for excluding subhaplogroup status K, U1b, U2e, U3, U4′9, U5b, and U6, so that U5a status would be most probable. In fact, mutation T15565C has been detected sporadically in some haplogroups, including haplogroup U5a (http://freepages.genealogy.rootsweb.ancestry.com/~ncscotts/mtDNA/GenBank%20Mutation%20Lists/hg%20U/mtDNA_hg_U_Mutation_Distribution.htm). The mtDNA lineage of patient UT9 is a J1c1 lineage, whereas the UT3 lineage is a specific J1c lineage not belonging to J1c1 but instead sharing the entire motif from the region 35–464 plus the infrequent mutation G8865A with the J1c lineage of GenBank accession number EU573192 (submitted by the company ‘Family Tree DNA’); the lack of G11719A likely represents an oversight. The mtDNA of patient UT6 is of non-European ancestry; this profile belongs to the East Asian haplogroup F3 as inferred from the rare combination 249del and A3434G.

Finally, the reported mtDNA variation of the patients T8 and UT4 is of mosaic nature. Namely, all mutations except for 309+C in region 35–464 match sequence no. 19 from ref. [41], which in particular encompass the rare mutation pair T279C, C285T that (in combination) is absolutely specific to haplogroup U1b. The coding-region variation, however, perfectly matches haplogroup K1a1b1 members. The most parsimonious explanation for this puzzling pattern is sample mix-up resulting in an inadvertent exchange of the amplicon for the control-region fragment. Similarly, patient T8 is assigned a mixed variation pattern, where the control-region fragment unambiguously indicates a haplogroup I lineage and the coding-region fragments point to haplogroup U (A11467G) instead of haplogroup I (with expected mutation G8251A).

The heteroplasmic variation is so immense and bizarre that it cannot reflect natural variation: most seeming heteroplasmies cluster in quite narrow stretches of the amplified fragments, especially in 3356–3410. Such patterns are indicative of artefacts termed phantom mutations. The amount of DNA and the number of PCR cycles were in fact too high, which together with a low temperature for the primers would invite all kinds of artefacts [53]. Similarly inadvertent amplification and sequencing conditions (following a forensic protocol that was widely used at the time) led to an excess of seeming heteroplasmies in hair roots [54], which was later admitted and corrected by the authors [55].

In summary, nearly all the heteroplasmic variation in Tables 1 and 2 of Carew et al. [10] is very likely induced by suboptimal amplification and sequencing protocols and thus artefactual. In contrast, the reported homoplasmic variation does not exhibit any excess or unexpected mutations: by and large, it matches the natural variation in the general population. A few mutations were actually not recorded, either by systematic employment of a wrong reference sequence or through oversight. Moreover, two samples were contaminated or confused at the amplification steps as testified by two clear-cut cases of artificial recombination.

### Apparent Instabilities and Data Quality Assessment

Heteroplasmic-like patterns frequently show up in the electropherograms as a result of sequence background noise. Some of these artefacts occur within the same sequencing plate and are not reproducible when replicating the sequencing in different plates. For instance, Data S3 shows an example affecting position 220 in HVS-II in one plate that was not replicated in a second sequencing round carried out in a different plate. Some of these unspecific artefacts appear as arrays of positions (e.g. 16105-16305-16310). Many of these spurious changes did not constitute typical phantom mutations that would occur at well-known sites [29], [56]. We have also observed that several positions appear to be instable (with heteroplasmic-like patterns) in forward but not in reverse sequence electropherograms or *vice versa*. With single-strand reading there is a high risk of taking these artefacts as face value of real mtDNA instability.

We also detected a case of sample mix-up due to an erroneous labelling of samples: LL79 was initially labelled as LL80 and *vice versa*. Since these two samples belong to two different haplogroups (with different sets of diagnostic motifs in the control-region), the error could be easily detected: the mtDNA profile of 79LG was C16111T-A16220C-T16362C-T16519C-A263G-309+C-315+C (belonging to haplogroup HV), while the profile of its seeming counterpart 79LL (80LL in reality) was C16069T-T16126C-G16145A-T16172C-T16231C-C16261T-A73G-C150T-T152C-T195C-A215G-A263G-C295T-310+T-315+C-T319C-T489C-G513A (haplogroup J2a1a1).

This kind of ‘artefactual instability’ due to sample mixing could go unnoticed in case the two samples would share very similar variation (e.g. when they belong to the same narrow sub-haplogroup). Therefore, the best way to determine whether both samples belong to the same biological source or not is to genotype a set of autosomal markers as was carried out in the present study.

### Confirmed Mutation Instabilities in B-CLL Patients

We considered confirmed mtDNA instabilities those sequence patterns that could be replicated in the first round of sequencing analysis in both forward and reverse strands, and additionally in an independent laboratory by a different analyst. Moreover, granulocyte and the counterpart lymphocyte mtDNA profiles matched phylogenetically in all the cases. Identification analysis carried out by means of STR autosomal profiling (as commonly exercised in the forensic field; ), further corroborated the common biological source of each pair of samples (lymphocytes/granulocytes) taken from the patients analyzed in the present study.

**Figure 1:**
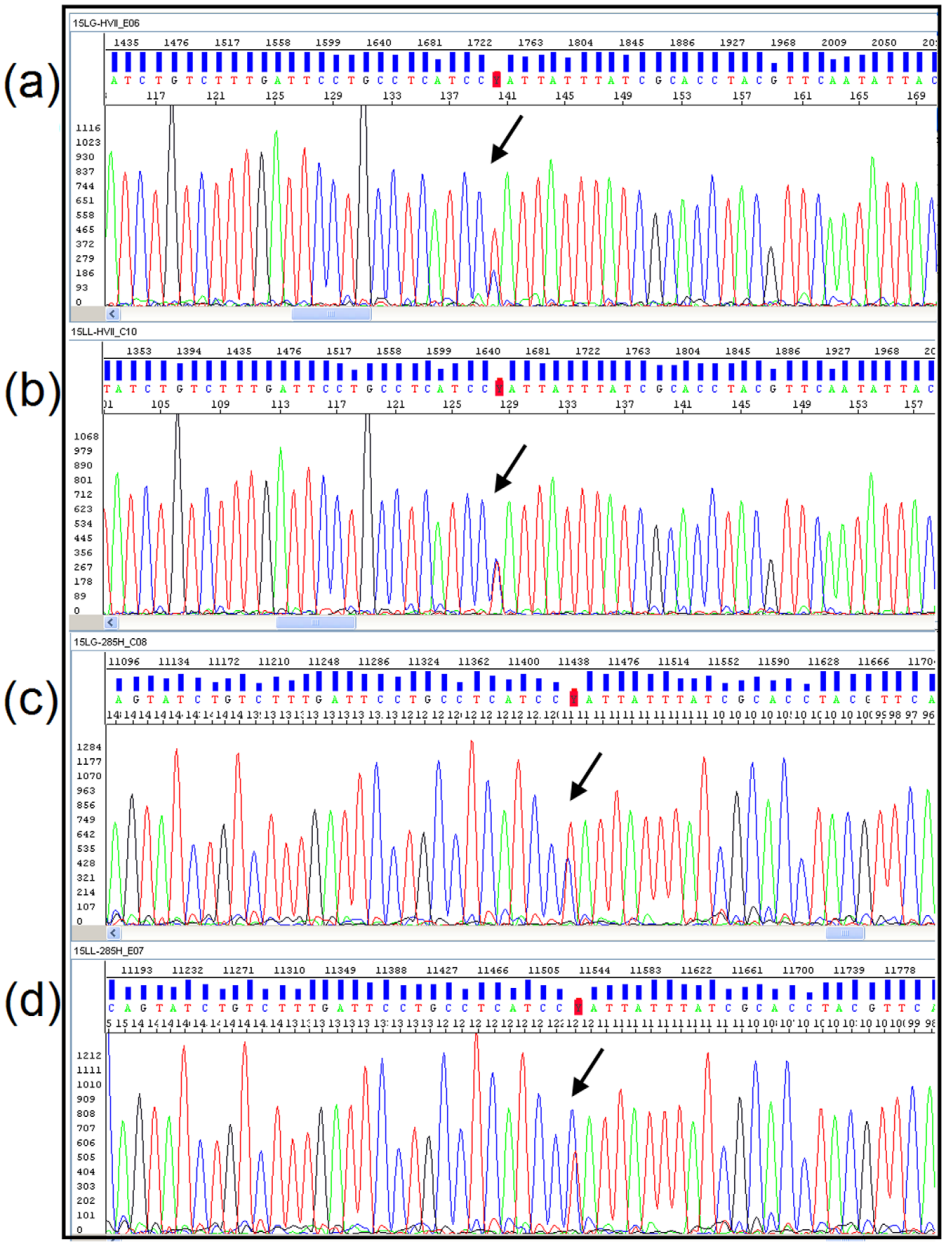
Example of mtDNA instability observed at position 152 in HVS-II; forward in granulocytes (a) and lymphocytes (b) and the reverse patterns in granulocytes (c) and lymphocytes (d).

We have detected instability-like patterns in a total of 20 patients (Table 3). Data S1 shows the full list of haplotypes obtained for the 146 patients analyzed in the present study. Figure 1 shows the electropherogam of a single example of instability whereas Data S4 shows the electropherograms for the full set of instabilities observed. Most of the instabilities (76%) appear associated with the homopolymeric C-stretch located in HVS-II around position 310. These instabilities are well known in forensic science. Further known hotspots affected by instabilities were positions 152 and 16093, the dinucleotide variation between 514 and 523, as well as the C-homopolymeric tract around position 570.

**Table 3:** Summary of the mutational differences found between granulocytes (G) and their counterpart lymphocytes (L).

NUCLEOTIDE POSITION	REGION	CHANGE (with respect to rCRS)	Frequency	Cell type
**16093**	MT-HV1	T→C	1 (homoplasmic)	G
		T→C>T	1 (heteroplasmic)	L
		T→T>C	1 (heteroplasmic)	G
		T→T≫C	1 (heteroplasmic)	L
**16224**	MT-HV1	T→C	1 (homoplasmic)	G
		T→C>T	1 (heteroplasmic)	L
**16235**	MT-HV1	A→G	1 (homoplasmic)	L
		A→G>A	1 (heteroplasmic)	G
**16270**	MT-HV1	C→T≫C	1 (heteroplasmic)	G
		C→T>C	1 (heteroplasmic)	L
**16302**	MT-HV1	A→A = G	1 (heteroplasmic)	G
		A→G>A	1 (heteroplasmic)	L
**16362**	MT-HV1	T→T>C	1 (heteroplasmic)	G
		T→C>T	1 (heteroplasmic)	L
**73**	MT-HV2	A→G	1 (homoplasmic)	L
		A→G≫A	1 (heteroplasmic)	G
**152**	MT-HV2, MT-OHR	T→T≫C	1 (heteroplamic)	L
		T→T>C	2 (heteroplasmic)	G
		T→T = C	1 (heteroplasmic)	L
**228**	MT-HV2, MT-OHR, MT-CSB1	G→A	1 (homoplasmic)	L
		G→A≫G	1 (heteroplasmic)	G
**303-309**	MT-HV2, MT-OHR, MT-CSB2	7C→8C	1 (homoplasmic)	L
		7C→8C>7C	2 (heteroplasmic)	G
		7C→8C = 9C	3 (heteroplasmic)	G and L
		7C→8C = 9C>7C	1 (heteroplasmic)	G
		7C→8C>9C	4 (heteroplasmic)	G and L
		7C→8C<9C	2 (heteroplasmic)	L
		7C→9C>10C	1 (heteroplasmic)	G
		7C→9C<10C	1 (heteroplasmic)	L
**514-523**		(CA)_5_→(CA)_5_>(CA)_4_	1 (heteroplasmic)	G
**568-573**		6C→7C	1 (homoplasmic)	L
		6C→8C>6C	1 (heteroplasmic)	G
		6C→9C	1 (heteroplasmic)	G
		6C→10C	1 (homoplasmic)	L
		6C→10C>9C	1 (heteroplasmic)	L

Most haplogroups characteristic of West Eurasia were present in our sample of B-CLL. The instabilities observed did not cluster in any particular haplogroup.

### *Testing for statistical association between haplogroup and clinical-pathological* variants and the incidence of mtDNA instabilities

The best *P*-value value for the potential association between the amount of instabilities and the haplogroup status was found to be for haplogroup H, (*P*-value = 0.0024; Chi-square). Adjustment for multiple testing either using Bonferroni or FDR test (adjusted α = 0.0022 for both tests) indicates a lack of statistical association between the presence of instabilities with the HG status and the clinical-pathological variants (Table 2).

## Discussion

Our experimental design explored the accumulation of mutations in two different cell lines that are biologically very closely related: both kinds of cell lines have their origin in single pluri-potent stem cells, so that the differences that could arise in the mtDNA of these two cells would be due mainly to the tumor condition of the lymphocyte line and not predominantly to somatic differences that arose during the differentiation process of these cell lines. We have detected a total of twenty cases carrying mtDNA instabilities, most of them affecting well-known hotspots in the mtDNA genome. This lends additional support to the hypothesis formulated in Vega et al. [16]; briefly, mtDNA instability occurs predominantly at natural hotspots and is at least in a first stage neutral to DNA function. We did not find any mtDNA instability that would follow a pathway in the mtDNA phylogeny and mimic a haplogroup motif (*contra* Linnartz et al. [57]).

Thus, an important amount of our analytical effort was devoted to the sequence quality and assessment of mtDNA instability. We only consider the existence of instability if it is confirmed in the forward, the reverse strands, in different DNA amplicons, and replicated in different laboratories. These aspects are particularly important in tumor studies since the use of relaxed criteria to assess instability can alter significantly results and conclusions. Despite the precautions for avoiding contamination, we detected an instance of sample mix-up that could have led to an erroneous interpretation of multiple instabilities (see above). Re-analysis of these samples uncovered the source of the error and fully ruled out the apparent evidence of instability.

In conclusion, instabilities observed in B-CLL patients seem to be neutral to DNA function and likely do not contribute to the tumor development.

We here advance some recommendations that would help to minimize erroneous interpretation of sequencing results in mtDNA studies in tumorigenesis:

- No instance of seeming heteroplasmy should be interpreted as real instability unless it is fully confirmed with forward and reverse sequencing.
- It is highly recommended to replicate positive results of instabilities in a second laboratory.
- Samples showing instability patterns (e.g. tumor and its counterpart ‘healthy’ control sample) should be genotyped for a set of autosomal markers (e.g. microsatellites) in order to preclude erroneous assignment of samples to an individual.
- Reverse and forward electropherograms for the instabilities found should be presented in manuscripts.
- Full mtDNA sequence results should be presented in the text [16] for the whole set of individuals analyzed rather than mere summary statistics that would only report the number of instabilities observed at individual positions [19].

## Supporting Information

Data S1 MtDNA sequencing results for the entire control-region of the mtDNA genome in 146 pairs of samples of granulocytes (HG or LG) and lymphocytes (HL or LL) obtained from B-CLL patients.
(0.05 MB XLS)

Data S2 LR values for the identification of the common biological source for those pairs of tumor/non-tumor samples showing mtDNA instabilities.
(0.02 MB XLS)

Data S3 Sequence electropherograms of the mtDNA heteroplasmic-like pattern at position 220 in HVS-II showing up as seemingly heteroplasmic in one plate but not replicated in a second round of sequencing analysis carried out in a different plate.
(0.07 MB DOC)

Data S4 Full set of sequence electropherograms showing the mtDNA instabilities detected in the present study. For each pairs of samples (indicated right below each tetrad of electropherograms together with the description of the instability observed), we indicate the forward (top pair electropherogram) and the reverse (bottom pair of electropherograms) sequences.
(2.22 MB DOC)
