# Supplementary material for: High Mitochondrial DNA Stability in B-Cell Chronic Lymphocytic Leukemia
Source: PLoS One. 2009 Nov 18;4(11):e7902. doi: 10.1371/journal.pone.0007902 (PMC2775629; doi:10.1371/journal.pone.0007902)
Supplement: Data S3 — Sequence electropherograms of the mtDNA heteroplasmic-like pattern at position 220 in HVS-II showing up as seemingly heteroplasmic in one plate but not replicated in a second round of sequencing analysis carried out in a different plate. (0.07 MB DOC) [file pone.0007902.s003.doc]

**Figure S1.** Sequence electropherograms of the mtDNA heteroplasmic-like patterns at position 220 in HVS-II showing up as heteroplasmic in one plate but not replicated in a second round of sequencing analysis carried out in a different plate.
