# Supplementary material for: High Mitochondrial DNA Stability in B-Cell Chronic Lymphocytic Leukemia
Source: PLoS One. 2009 Nov 18;4(11):e7902. doi: 10.1371/journal.pone.0007902 (PMC2775629; doi:10.1371/journal.pone.0007902)
Supplement: Data S4 — Full set of sequence electropherograms showing the mtDNA instabilities detected in the present study. For each pairs of samples (indicated right below each tetrad of electropherograms together with the description of the instability observed), we indicate the forward (top pair electropherogram) and the reverse (bottom pair of electropherograms) sequences. (2.22 MB DOC) [file pone.0007902.s004.doc]

**Figure S3.** Full set of sequence electropherograms showing the mtDNA instabilities detected in the present study. For each pairs of samples (indicated right below each tetrad of electropherograms together with the description of the instability observed), we indicate the forward (top pair electropherogram) and the reverse (bottom pair of electropherograms) sequences.

| 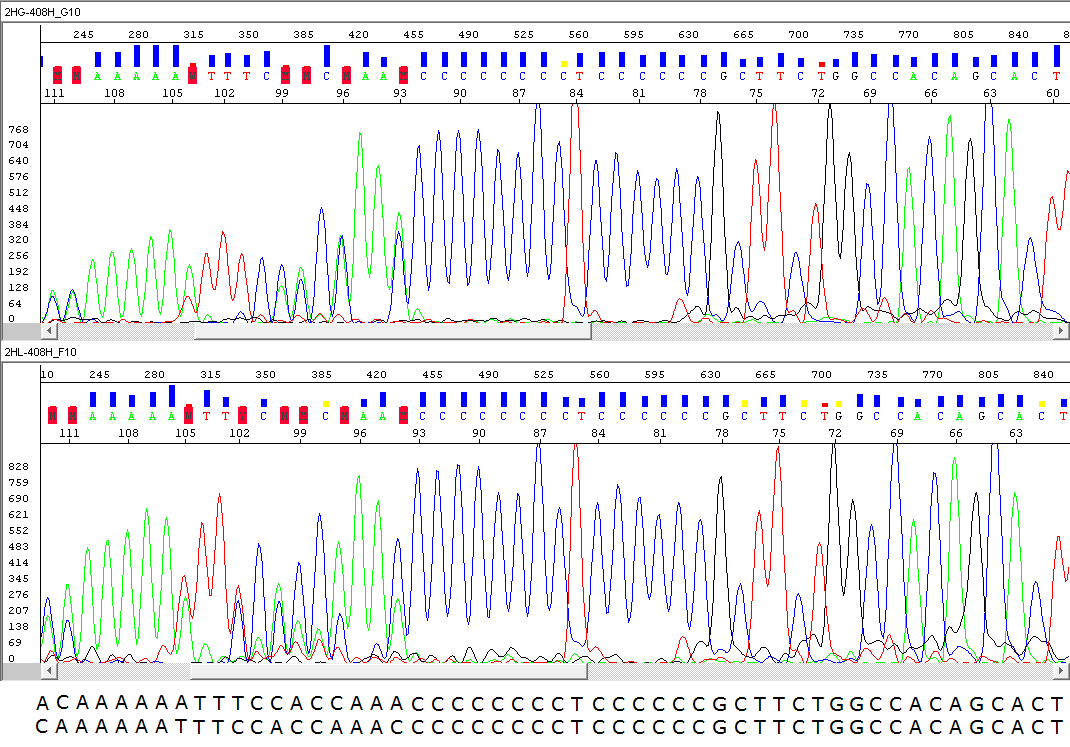 |  |
| --- | --- |
| 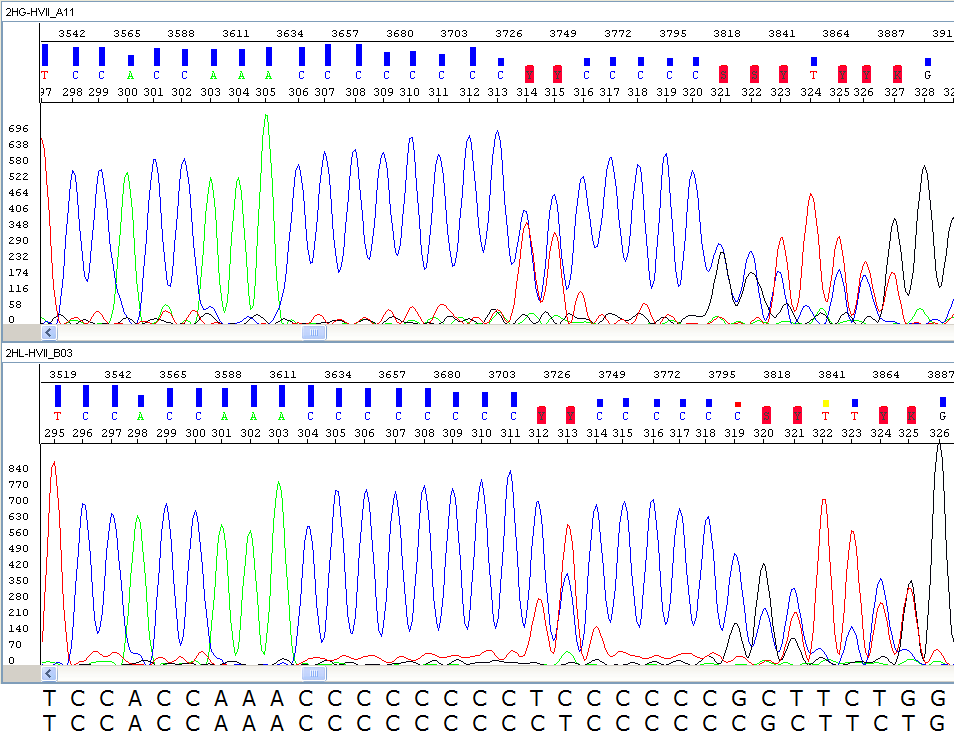 |  |
| 2HG and 2HL: 309+C=309+CC/309+CC>309+C |  |
| 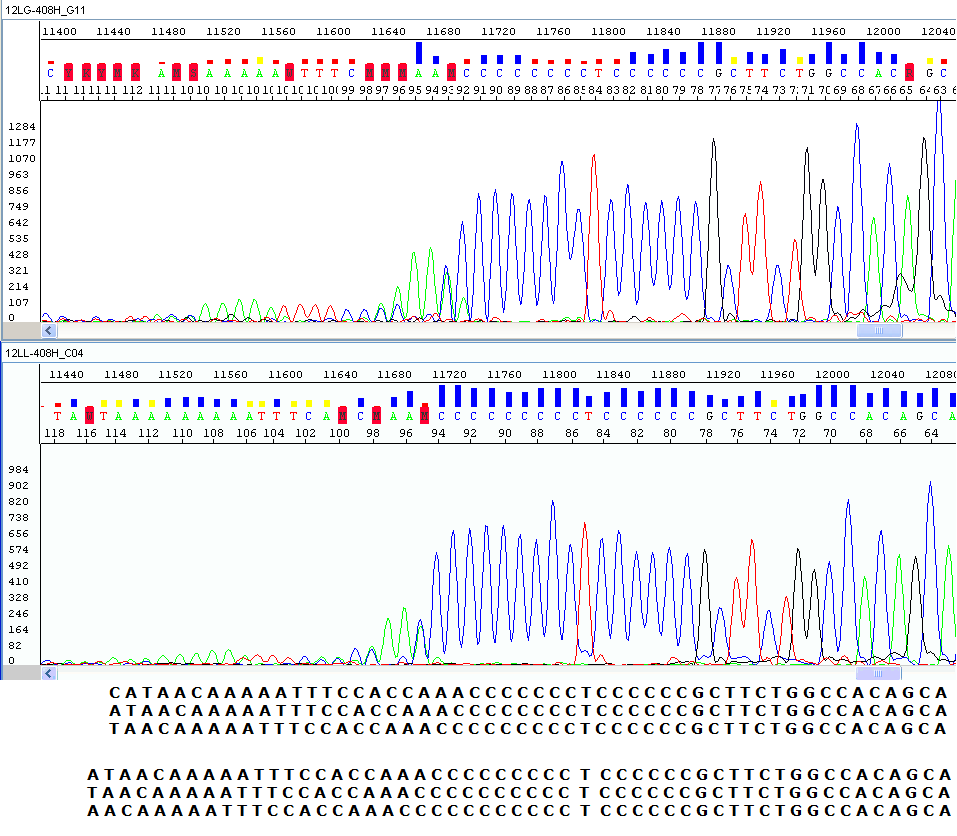 |  |
| 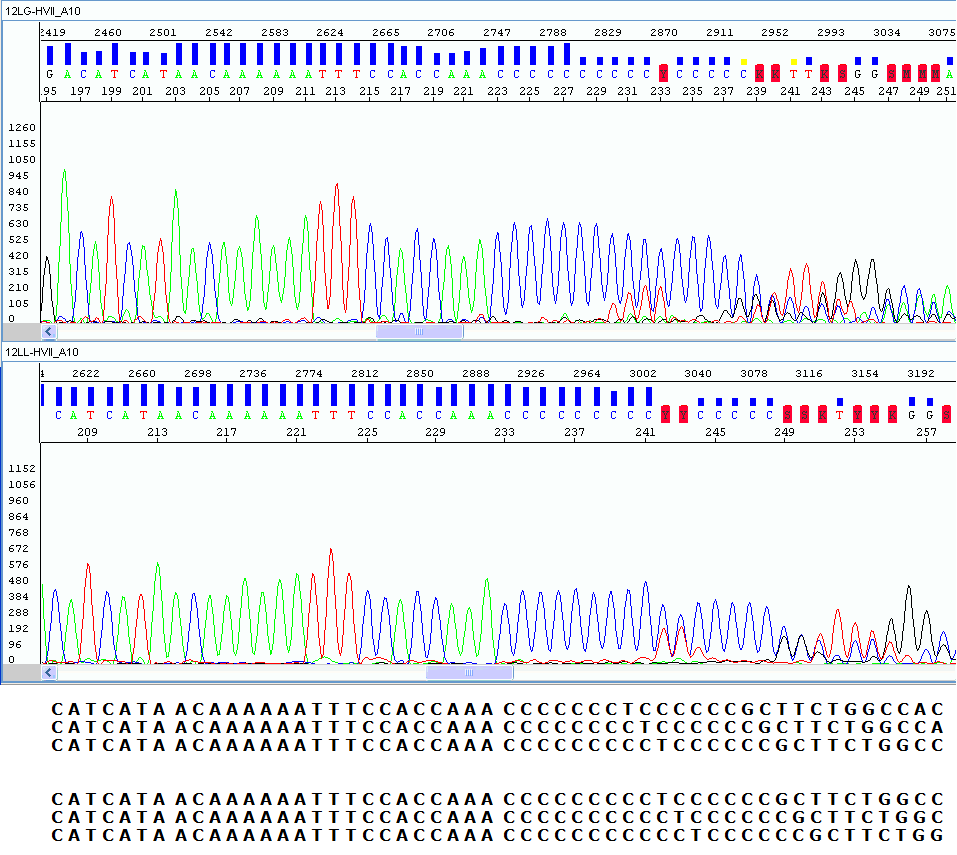 |  |
| 12LG and 12LL: 309+C=309+CC>rCRS/309+C=309+CC |  |
| 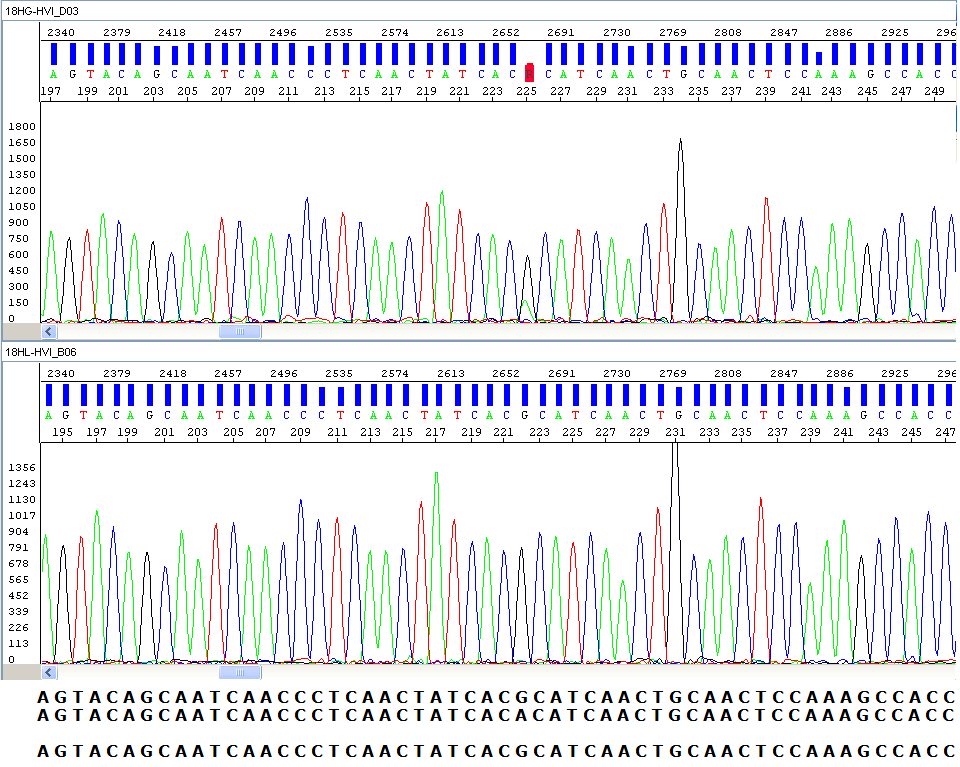 |  |
| 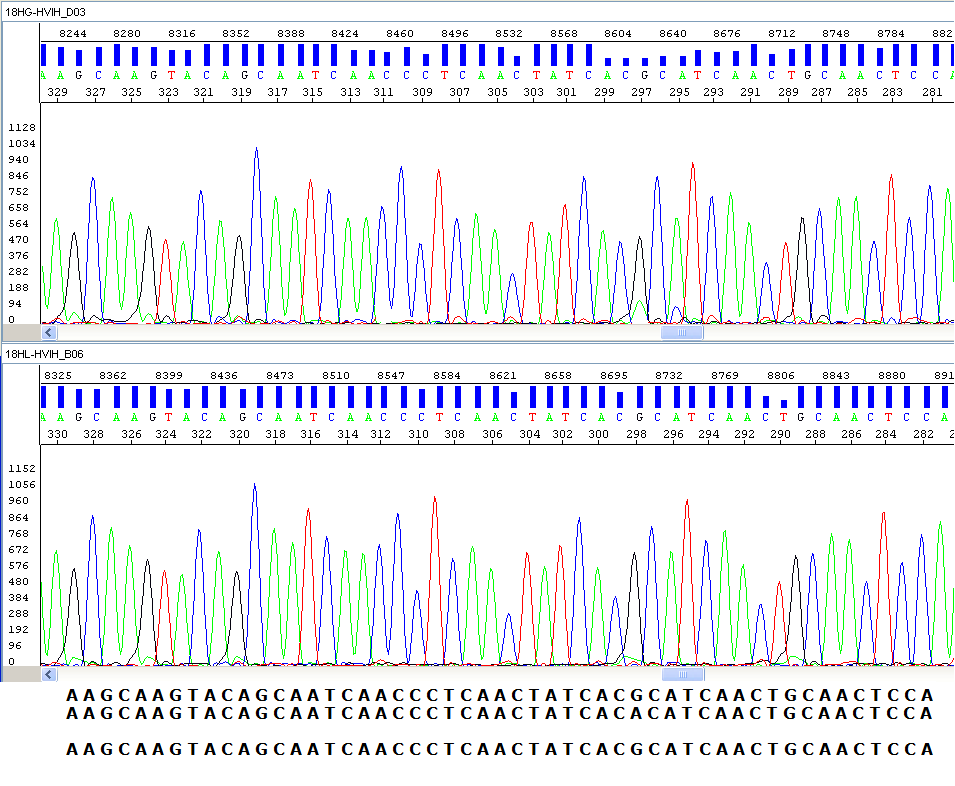 |  |
| 18HG and 18HL: 16235G>A/16235 |  |
| 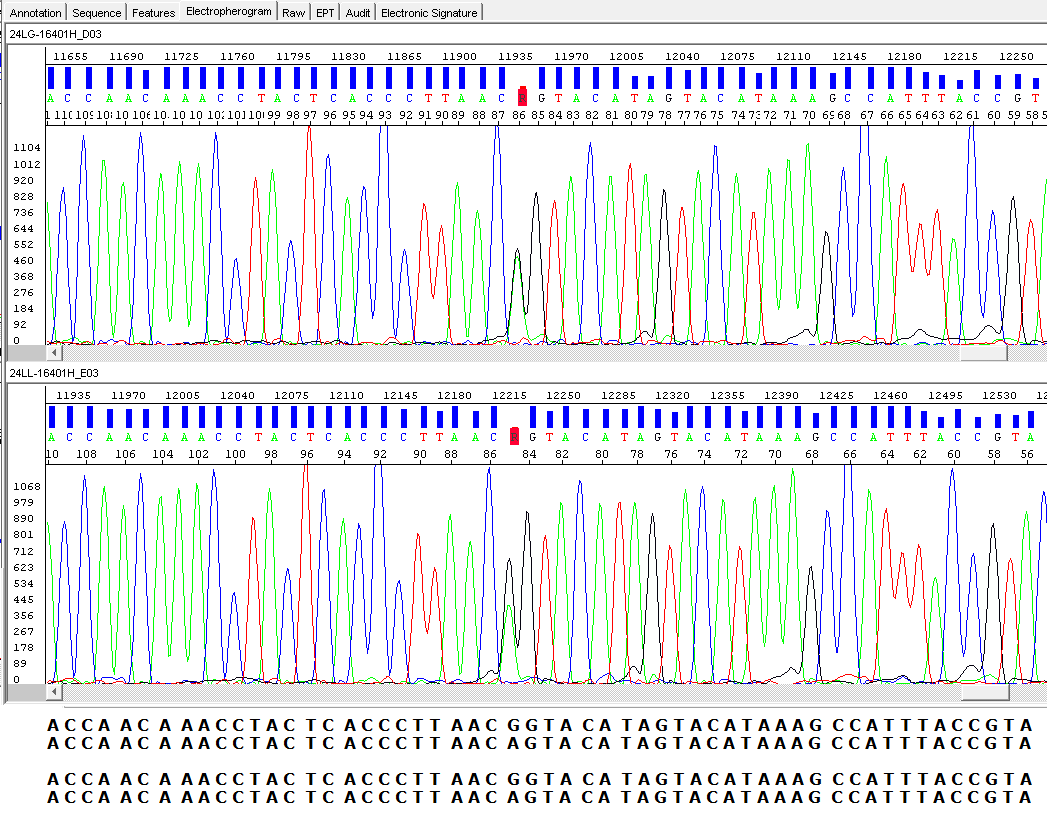 |  |
| 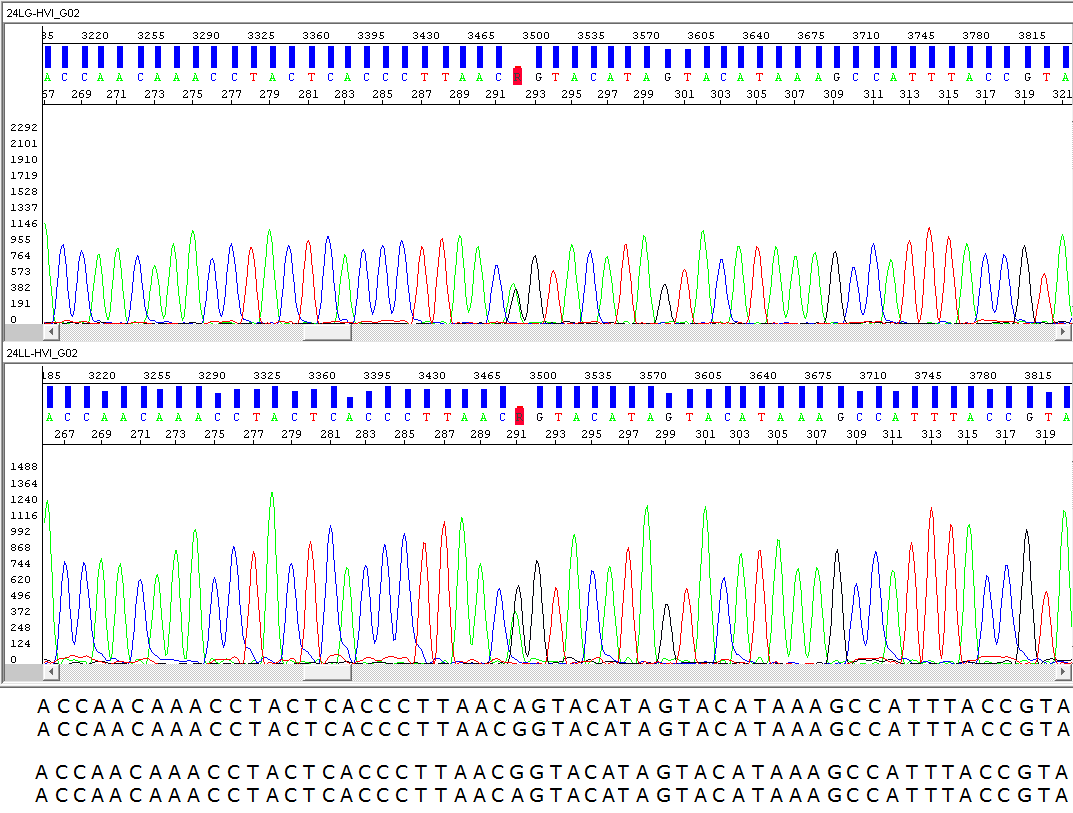 |  |
| 24LG and 24LL: 16302G=A/16302G>A |  |
| 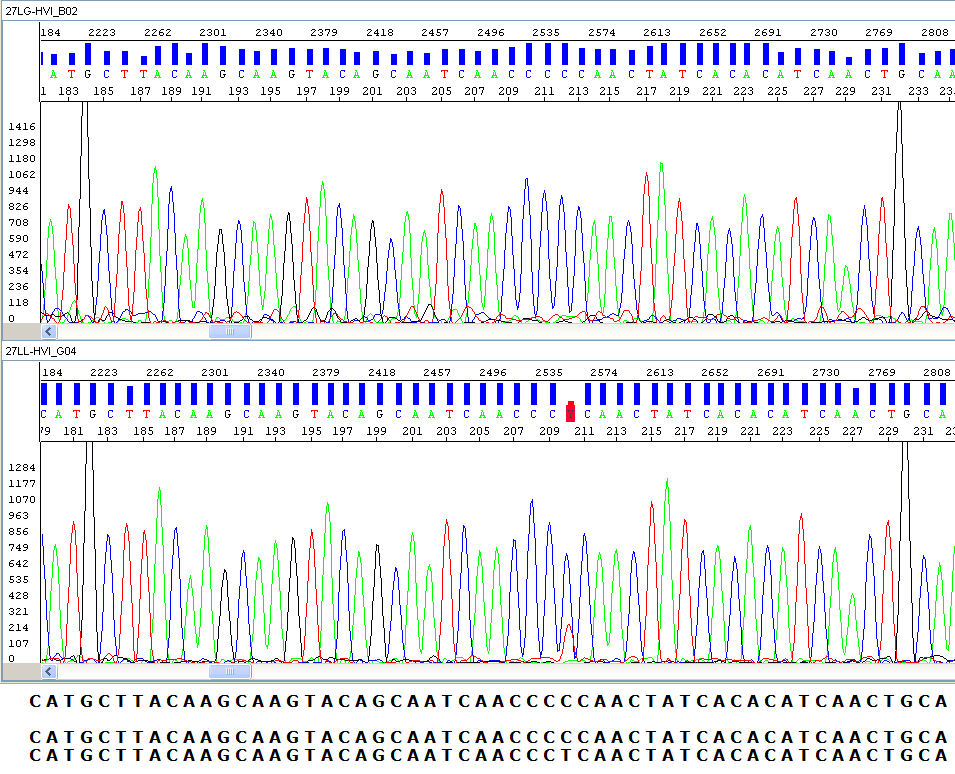 | |
| 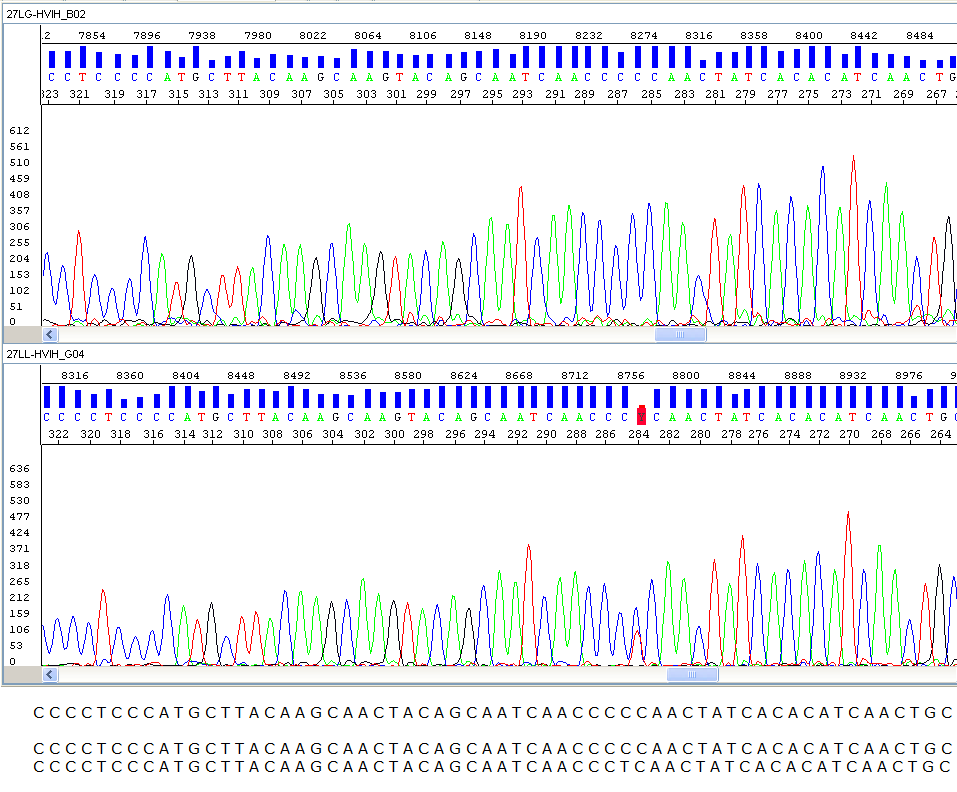 | |
| 27LG and 27LL: 16224/16224C>T | |
| 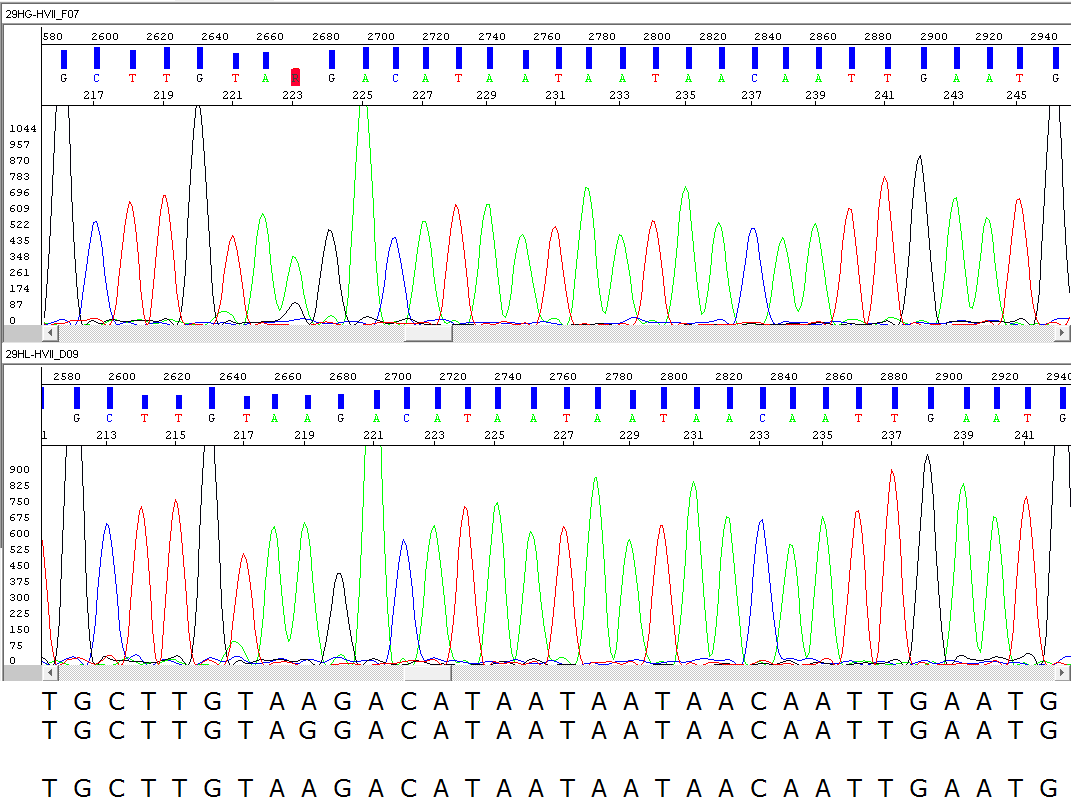 | |
| 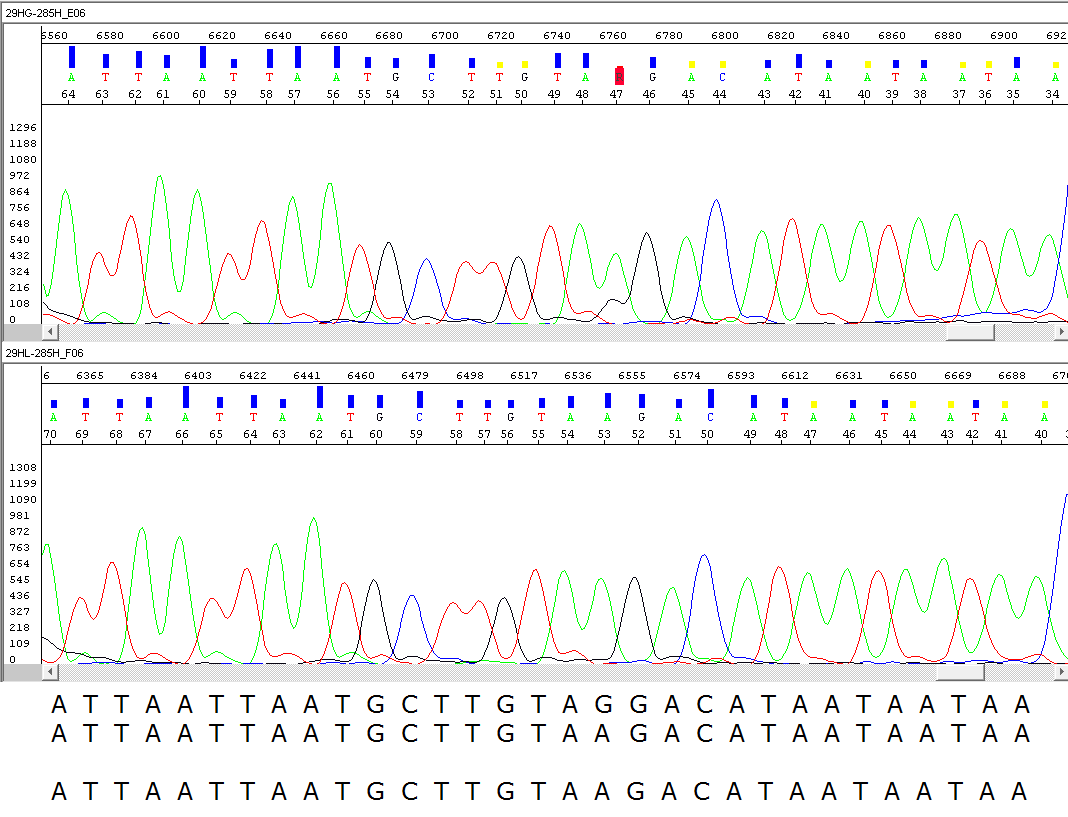 | |
| 29HG and 29HL: 228A>>G/228 | |
| 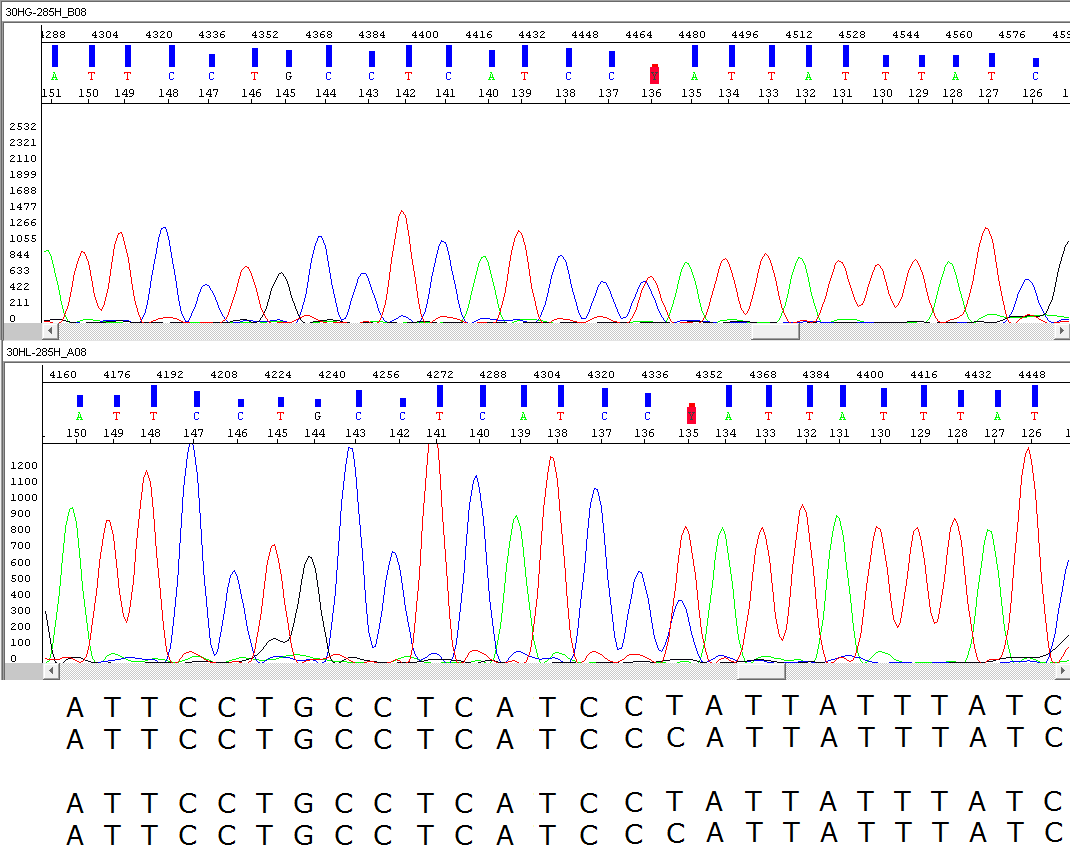 | |
| 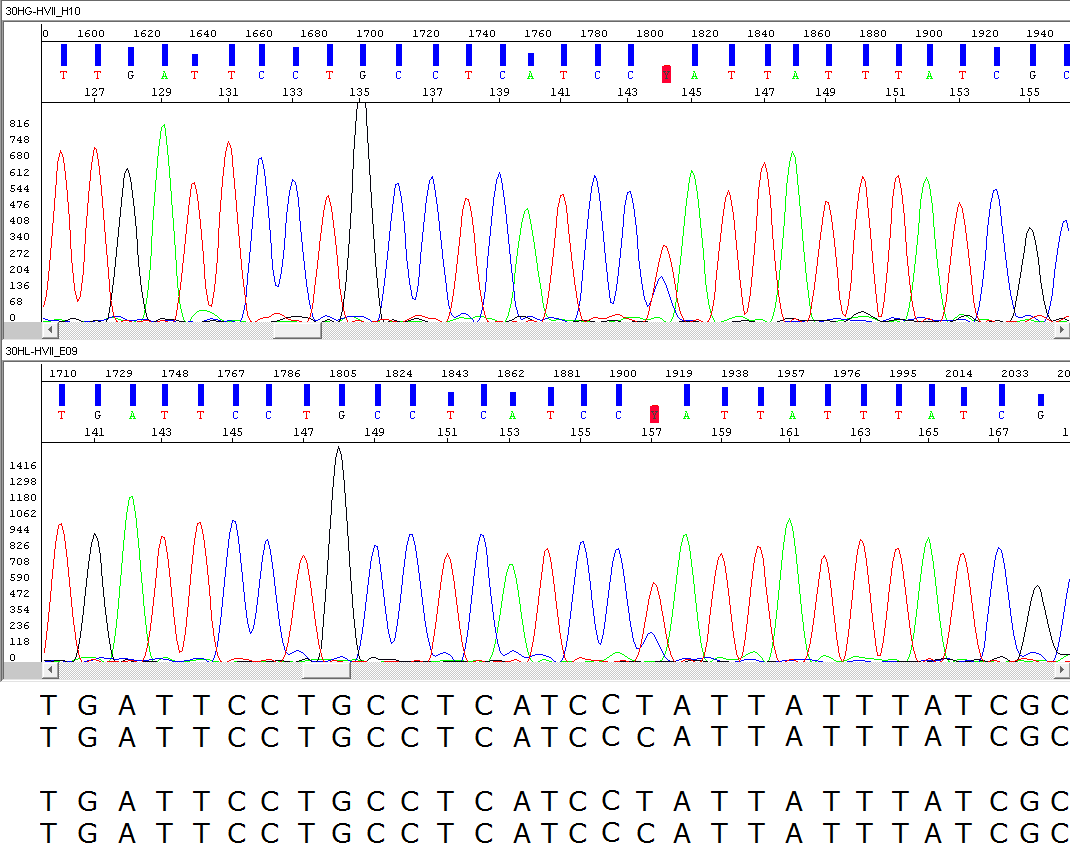 | |
| 30HG and 30HL: 152T>C/152T>>C | |
| 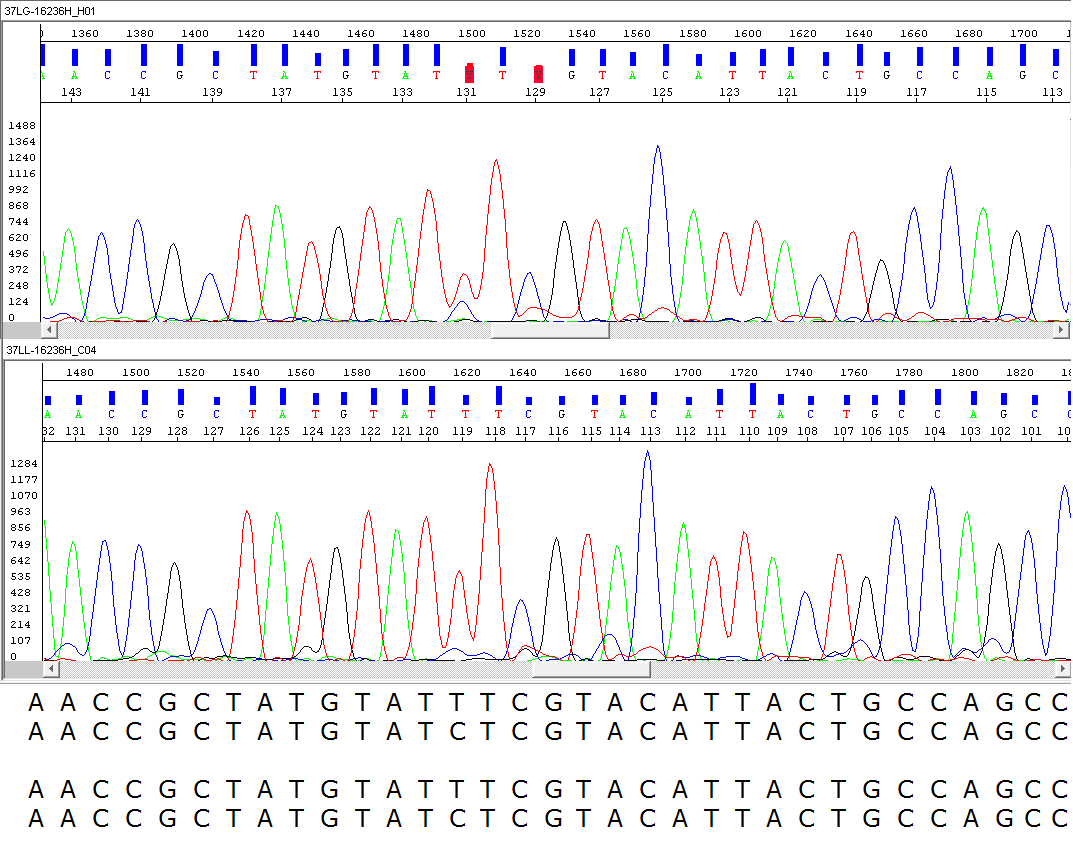 | |
| 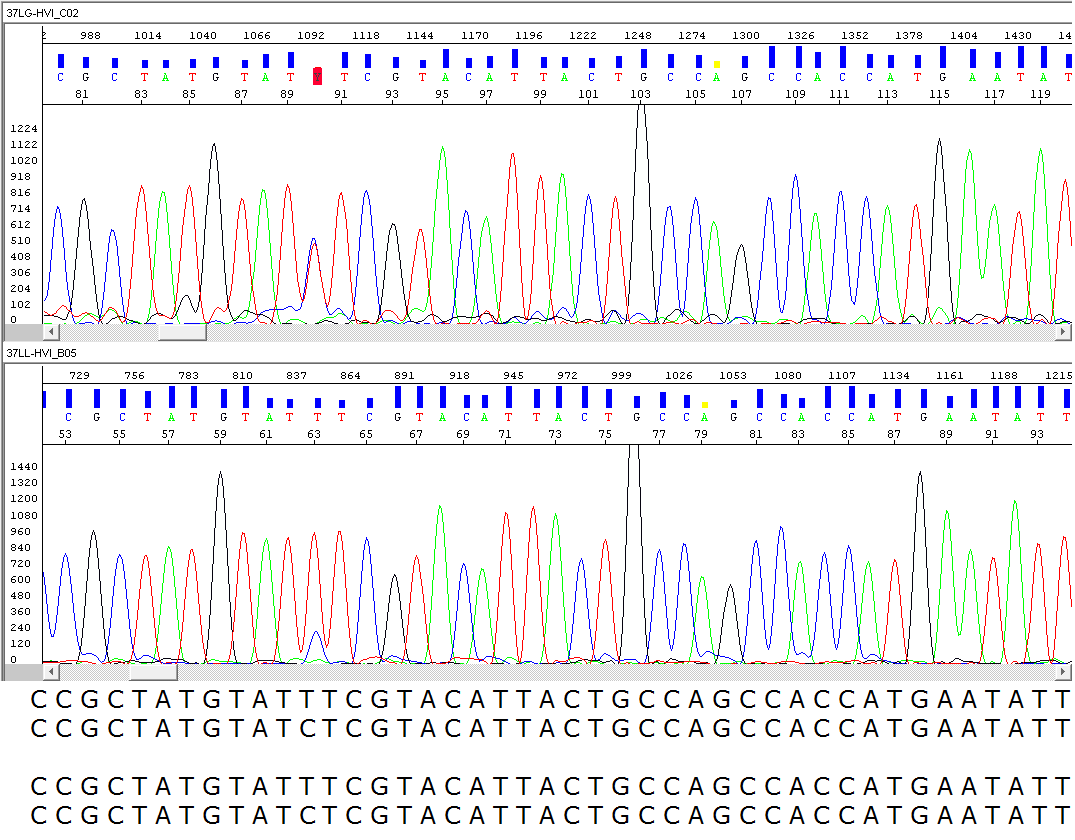 | |
| 37LG and 37LL: 16093T>C/16093T>>C | |
| 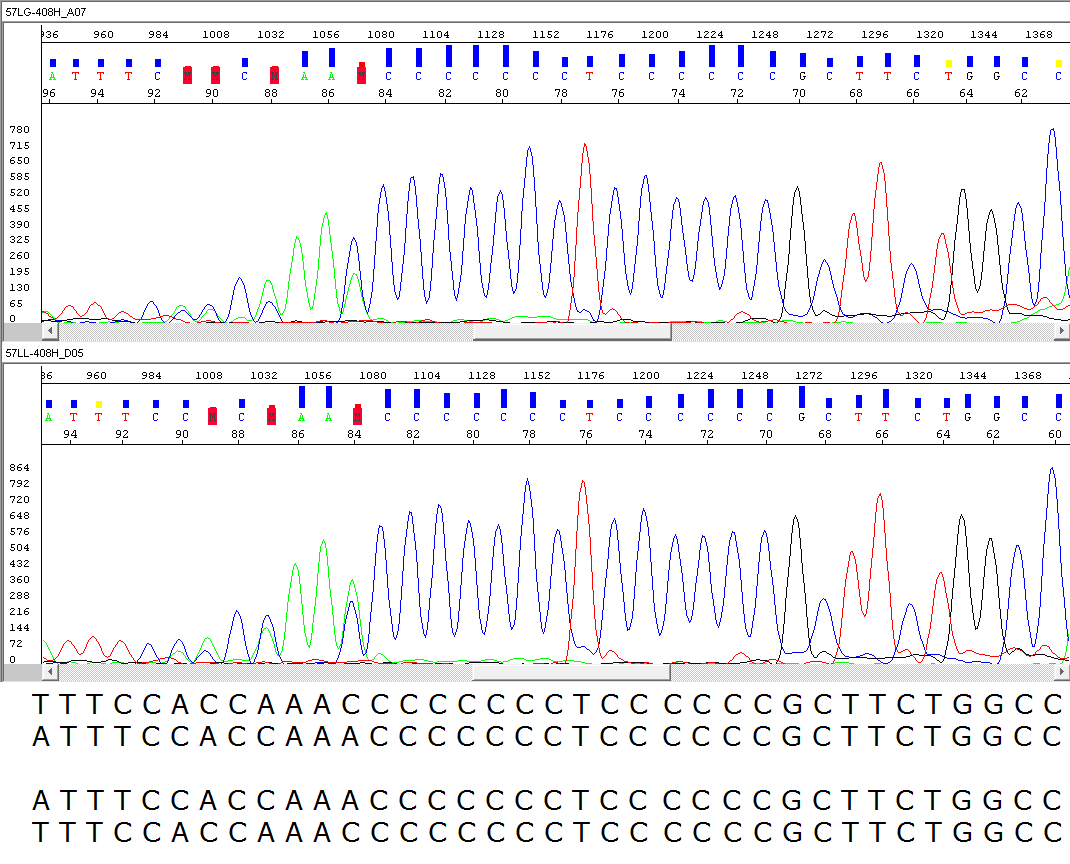 | |
| 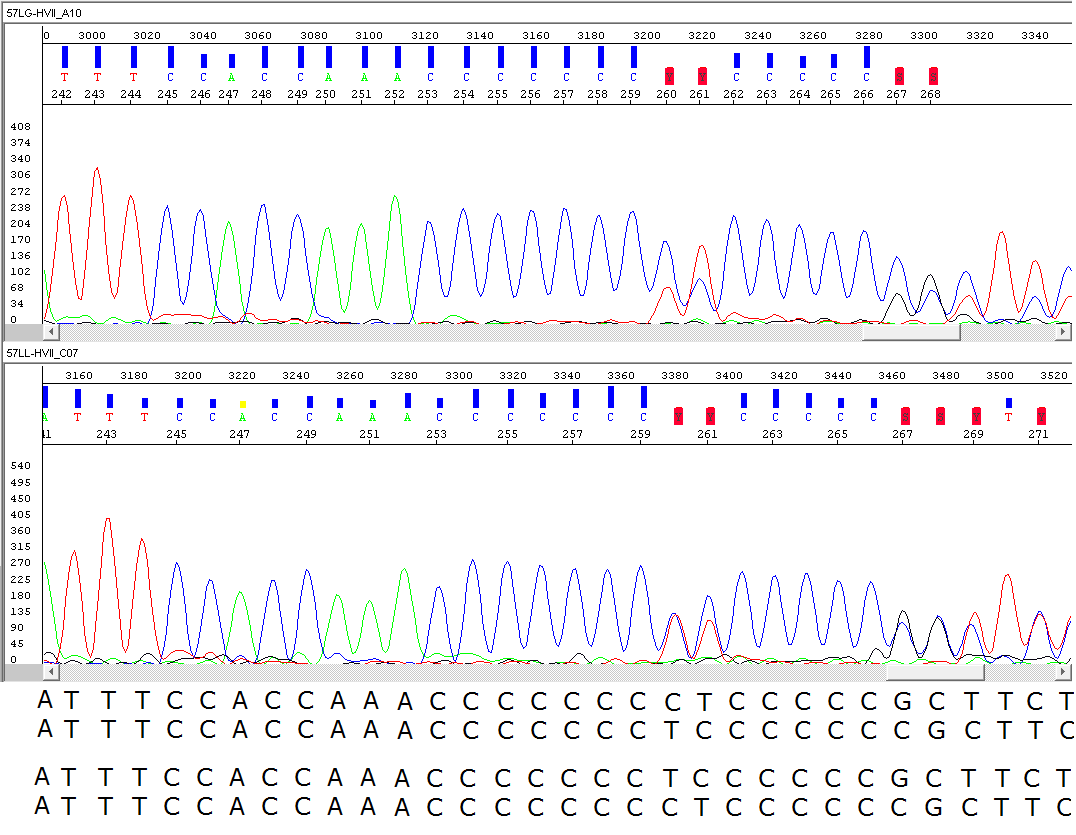 | |
| 57LG and 57LL: rCRS<309+C/rCRS>309+C | |
| 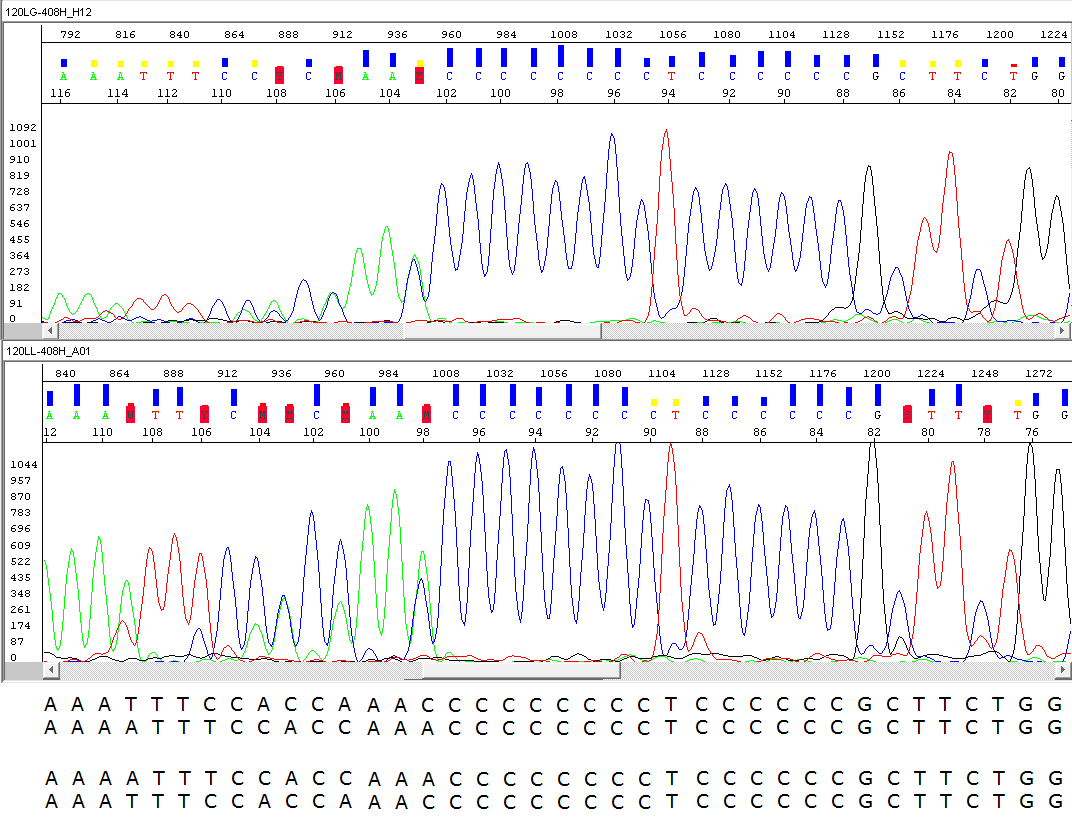 | |
| 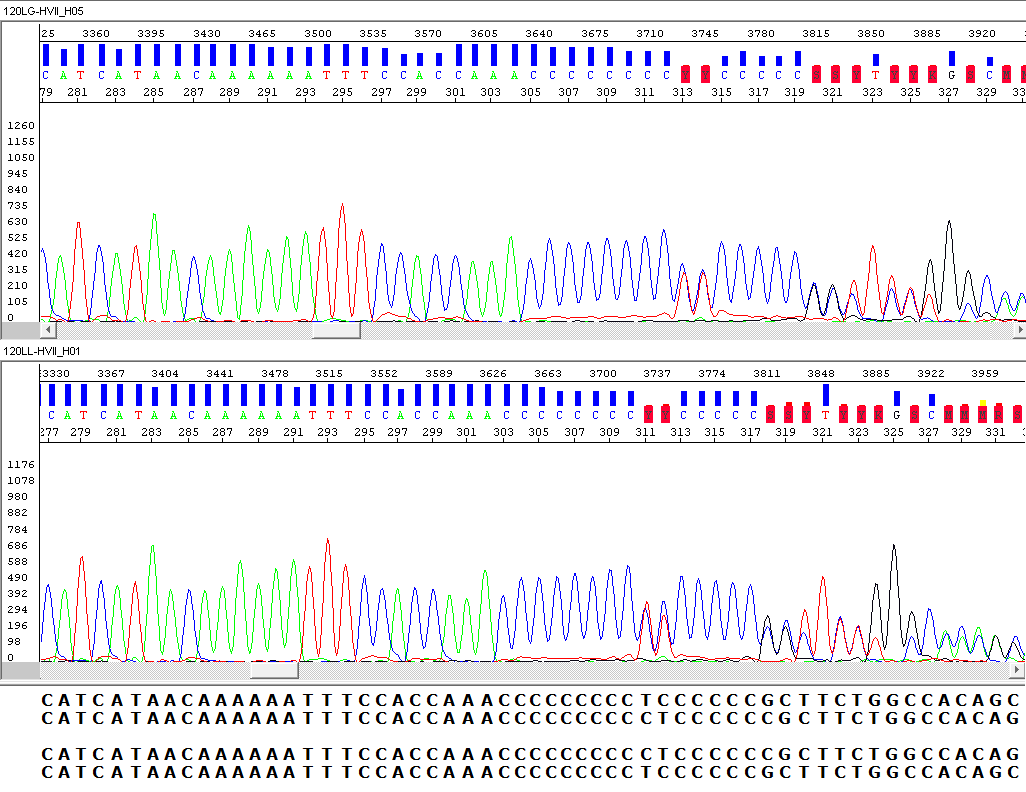 | |
| 120LG and 120LL: 309+C=309+CC/309+C>309+CC | |
| 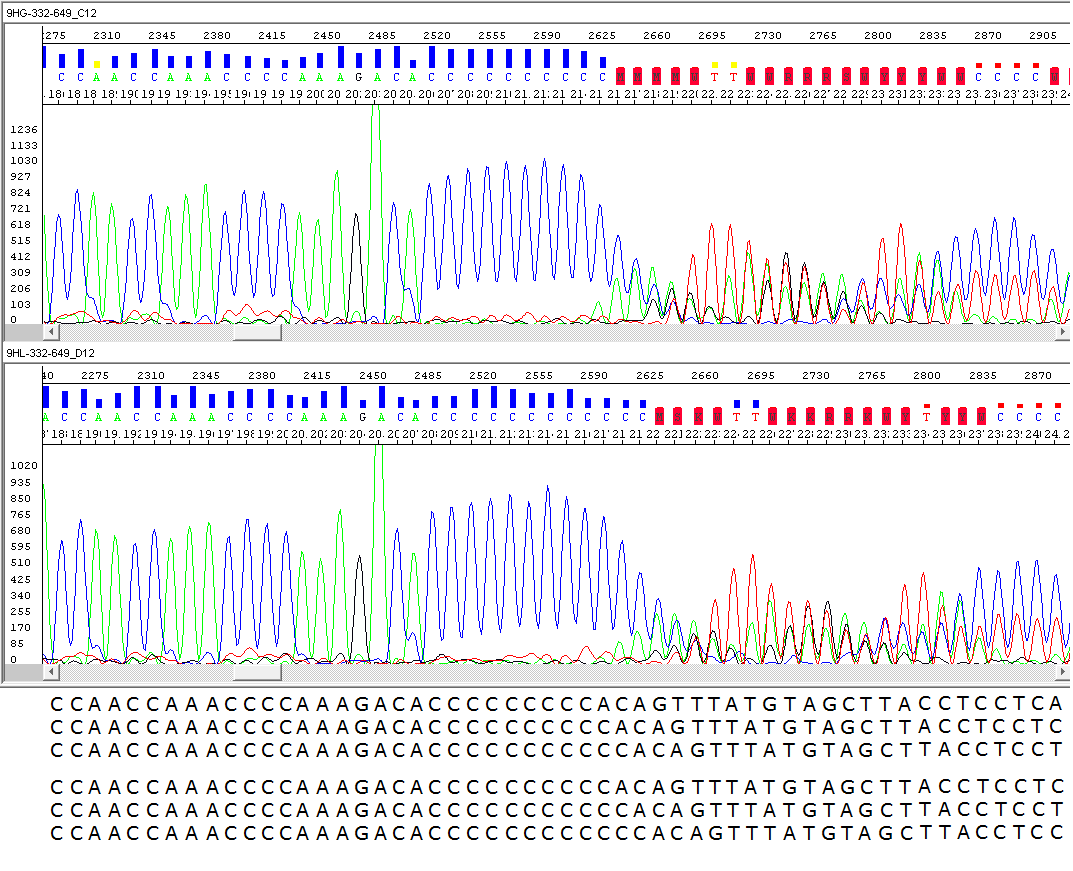 | |
| 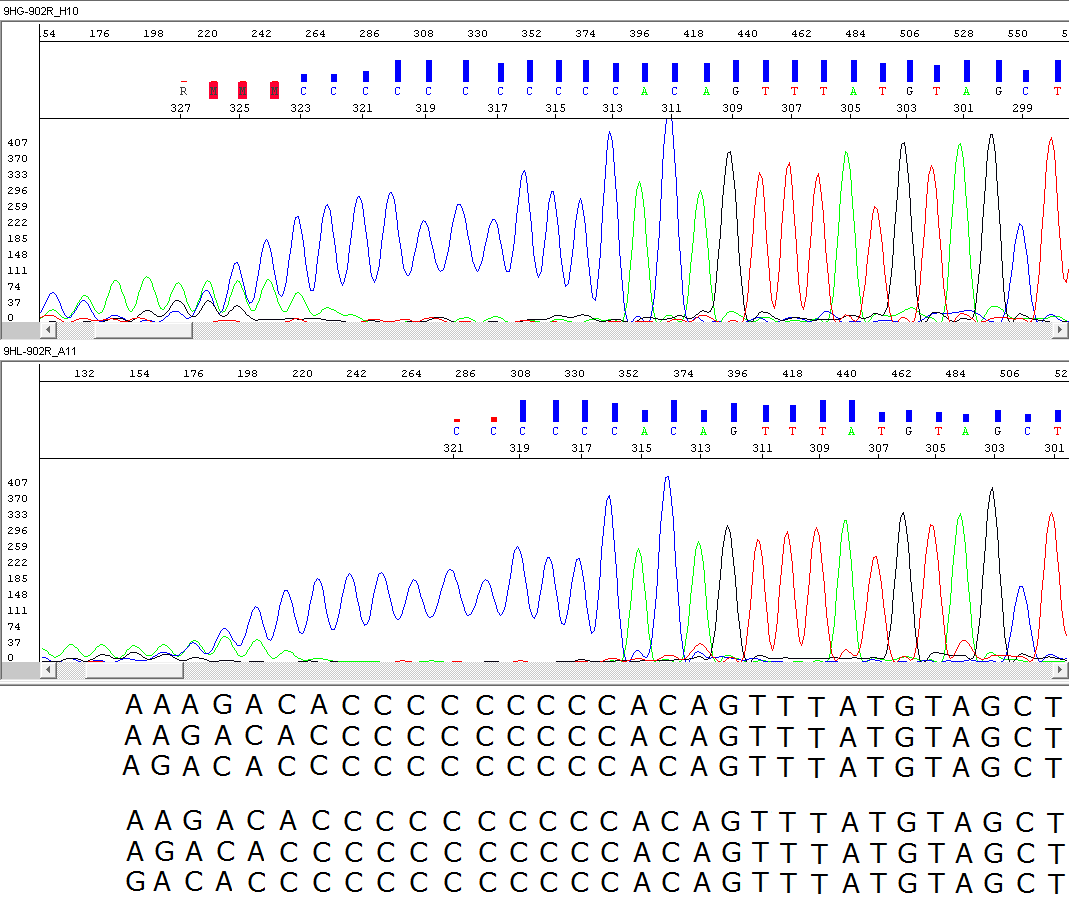 | |
| 9HG and 9HL: 573+3C/573+4C | |
| 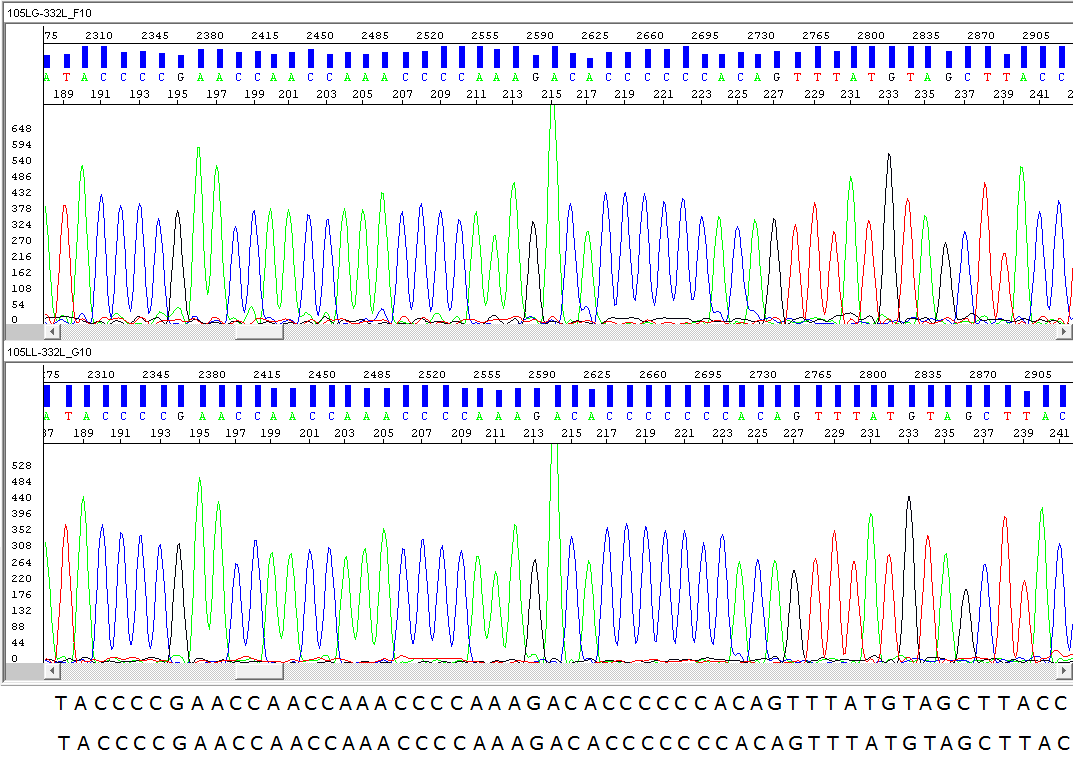 | |
| 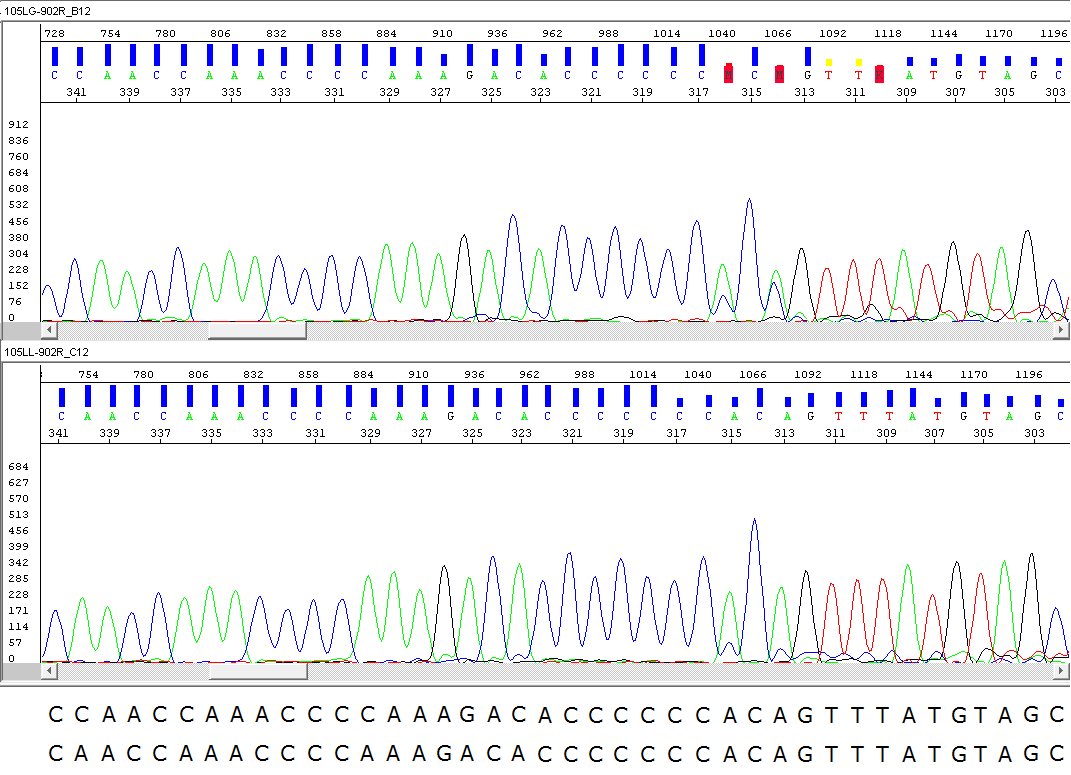 | |
| 105LG and 105LL: rCRS/573+C | |
| 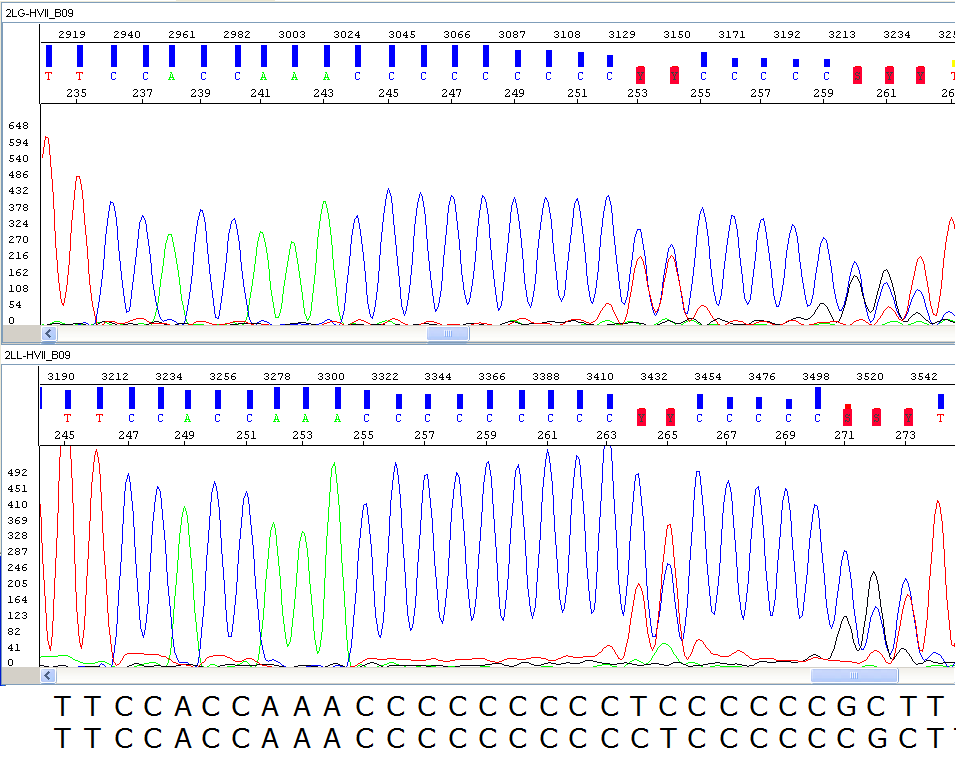 | |
| 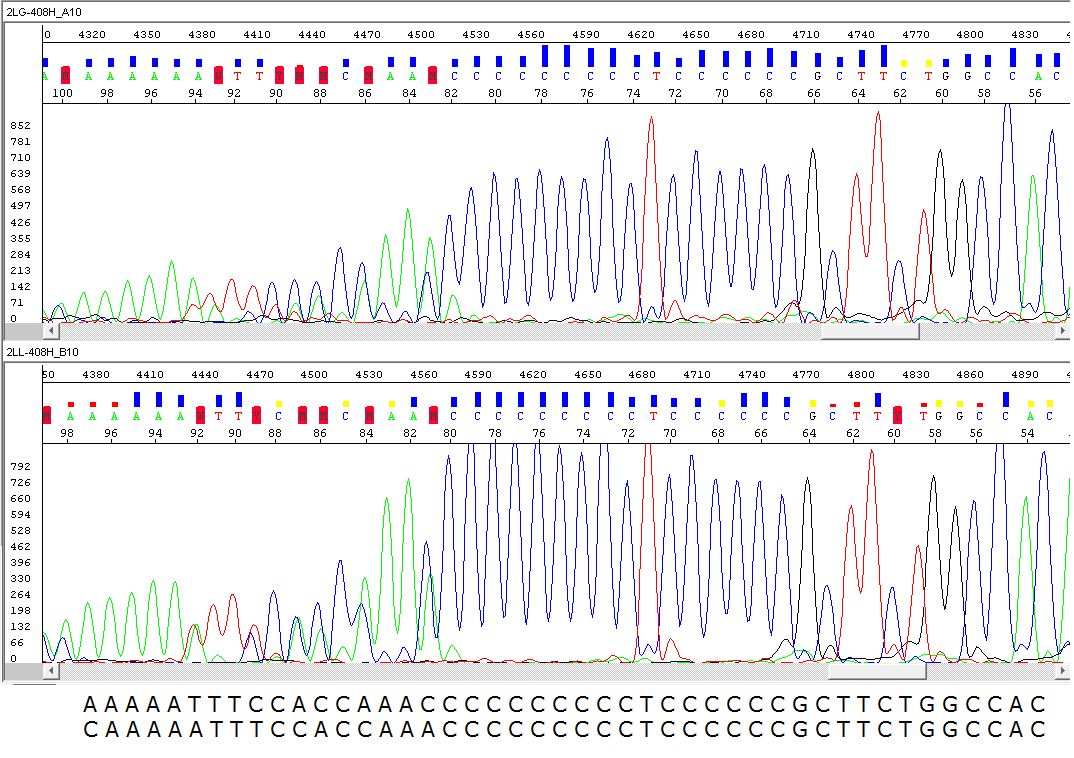 | |
| 2LG and 2LL: 309+CC>309+CCC/309+CCC>309+CC | |
| 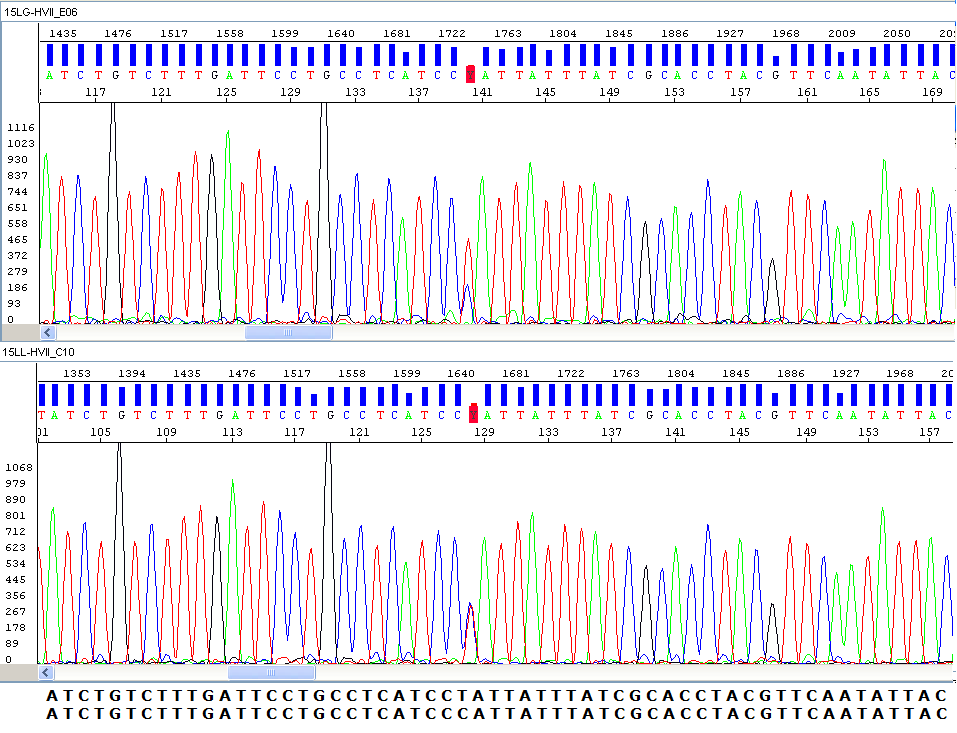 | |
| 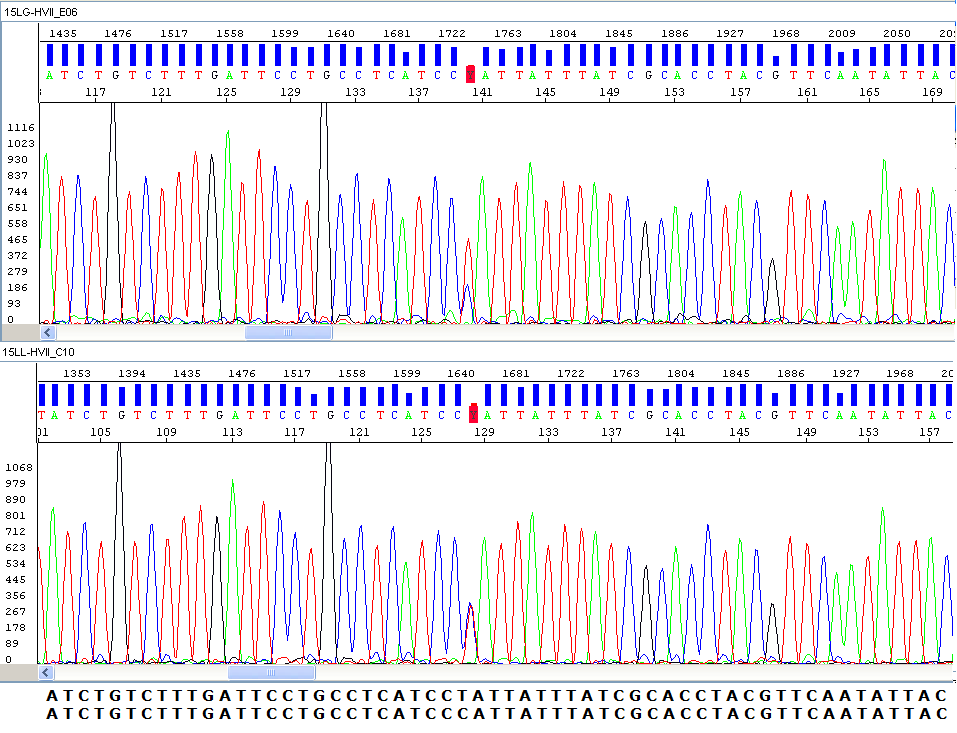 | |
| 15LG and 15LL: 152T>C/152T=C | |
| 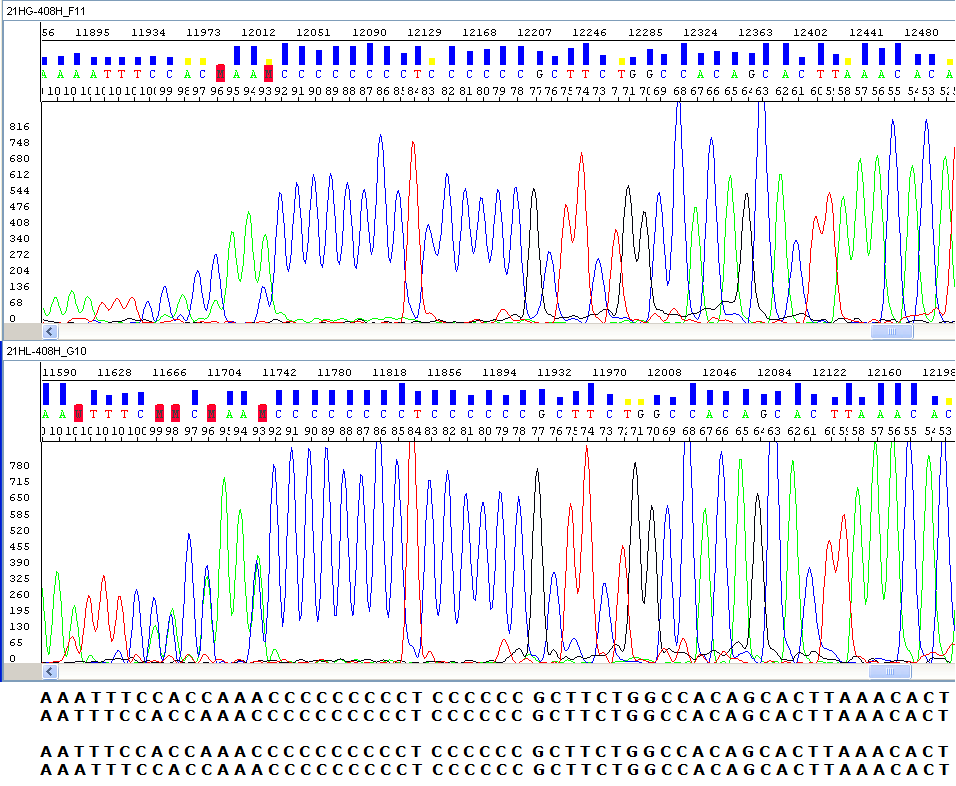 | |
| 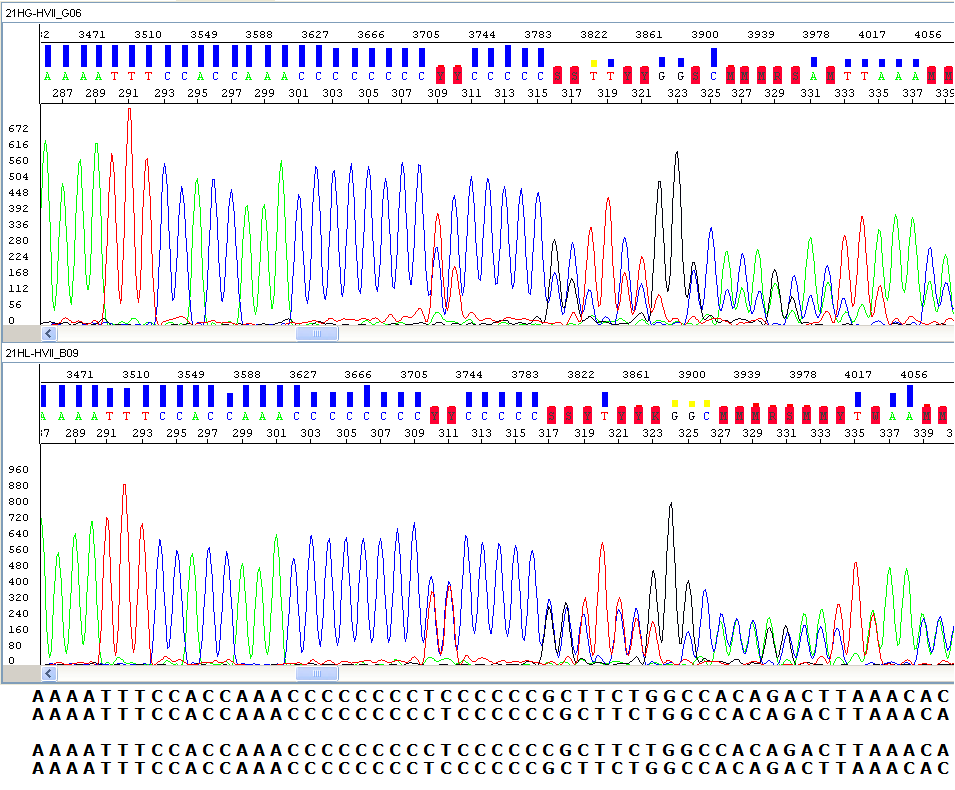 | |
| 21HG and 21HL: 309+C>309+CC/309+C=309+CC | |
| 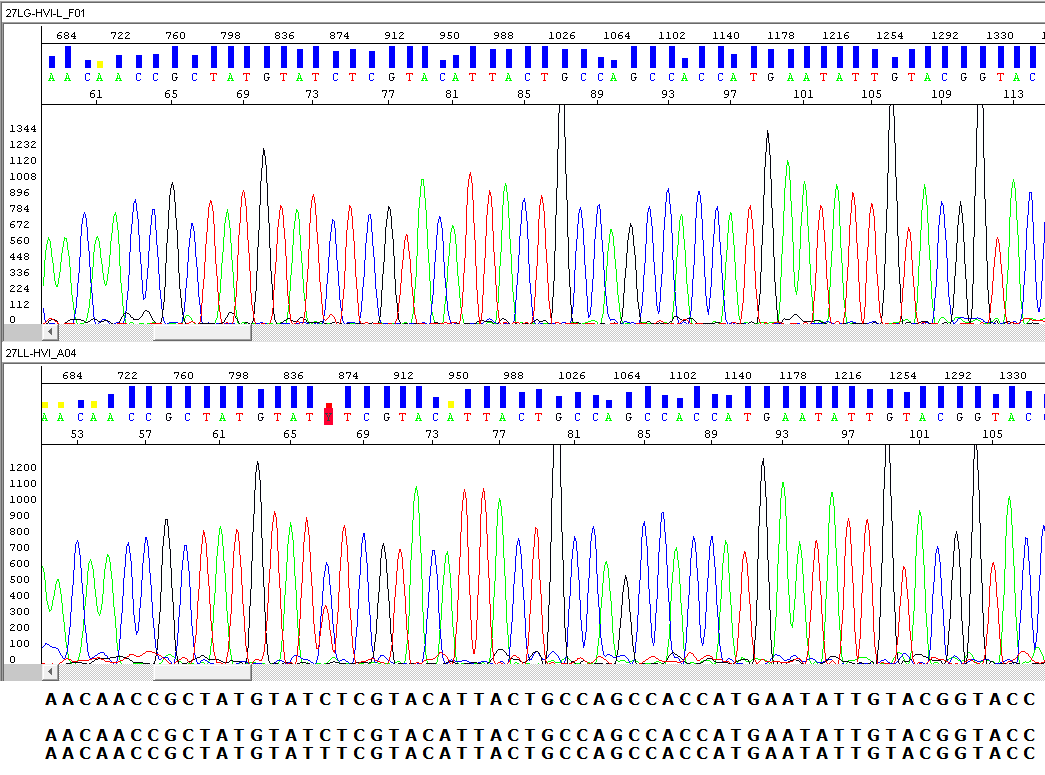 | |
| 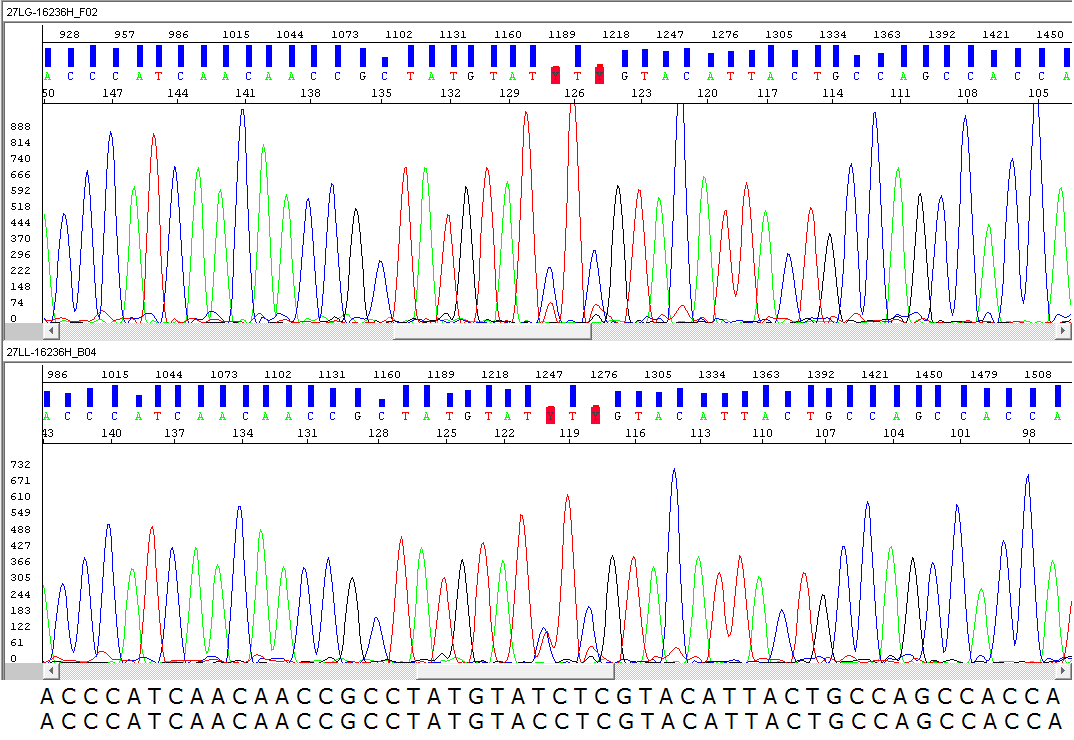 | |
| 27LG and 27LL: 16093/16093C>T | |
| 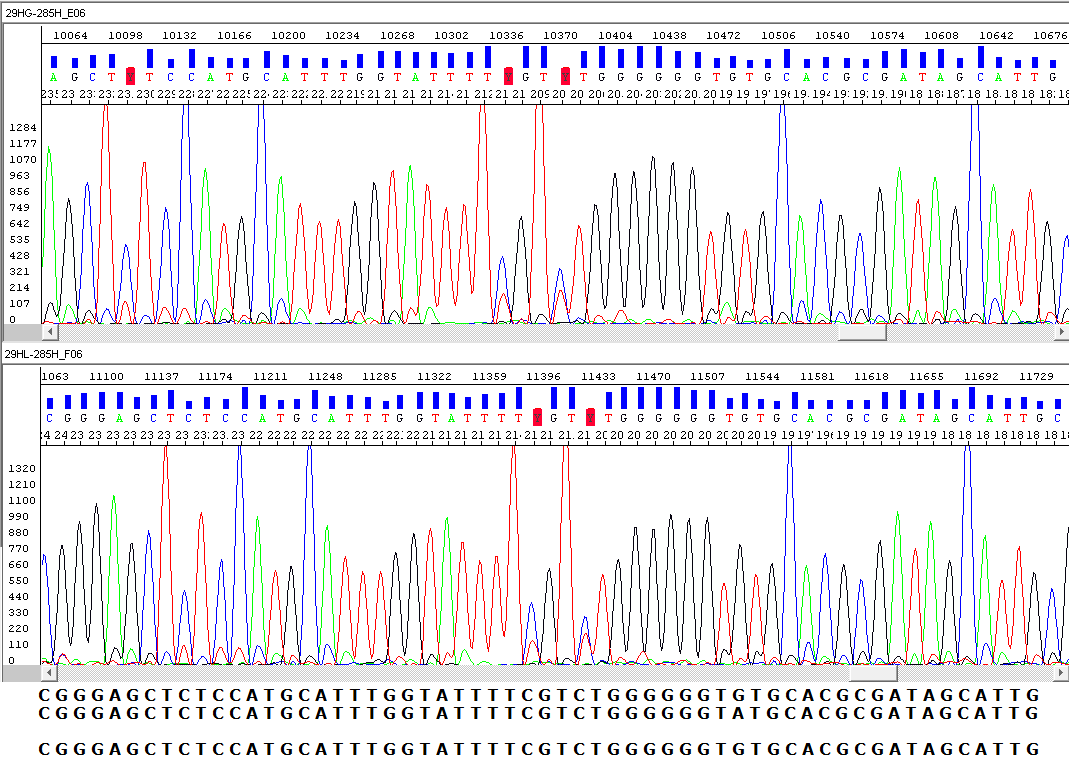 | |
| 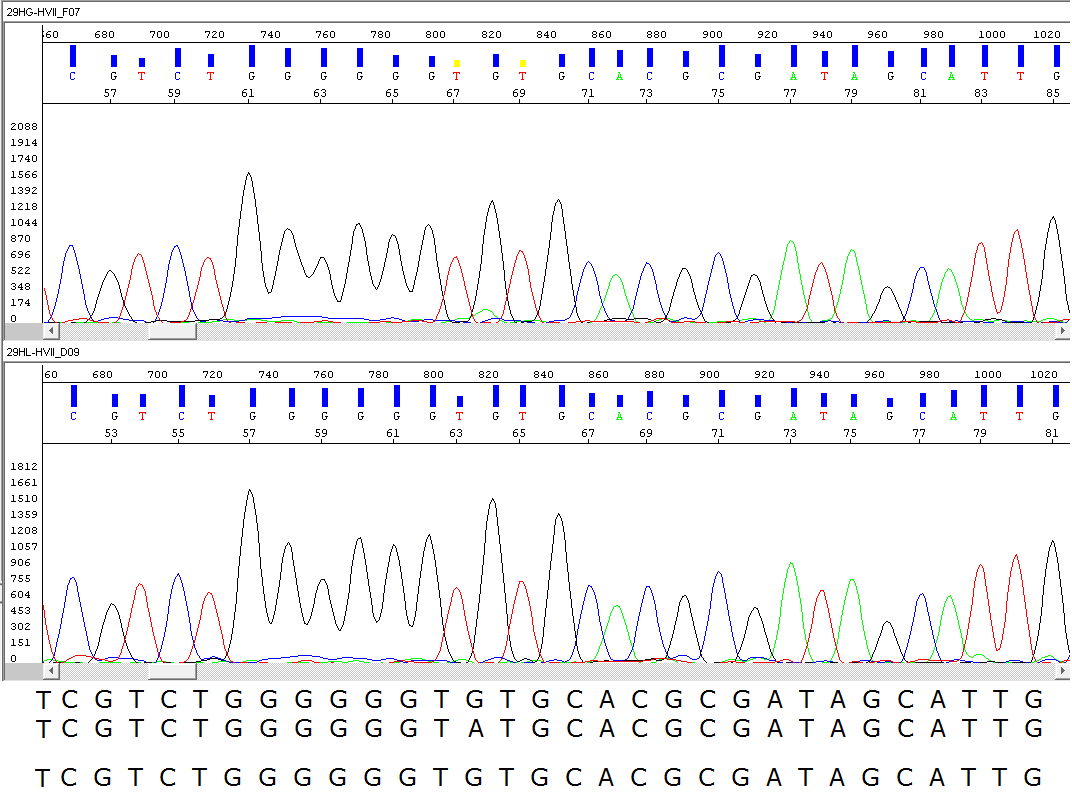 | |
| 29HG and 29HL: 073G>>A/073 | |
| 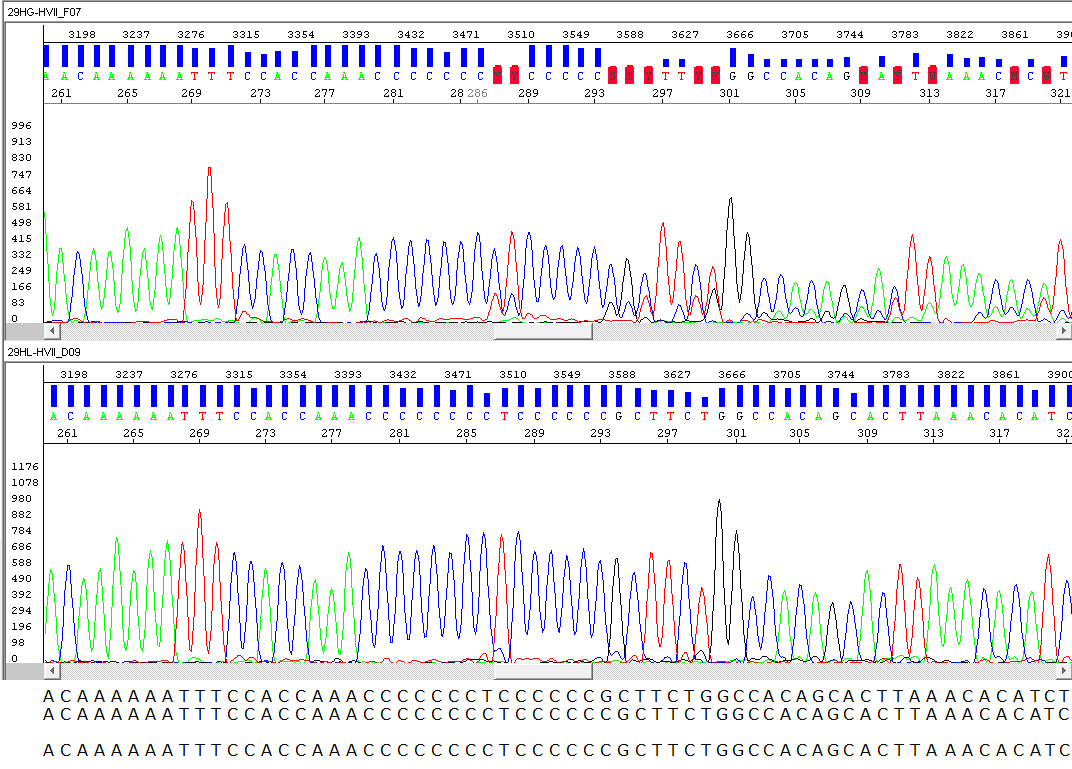 | |
| 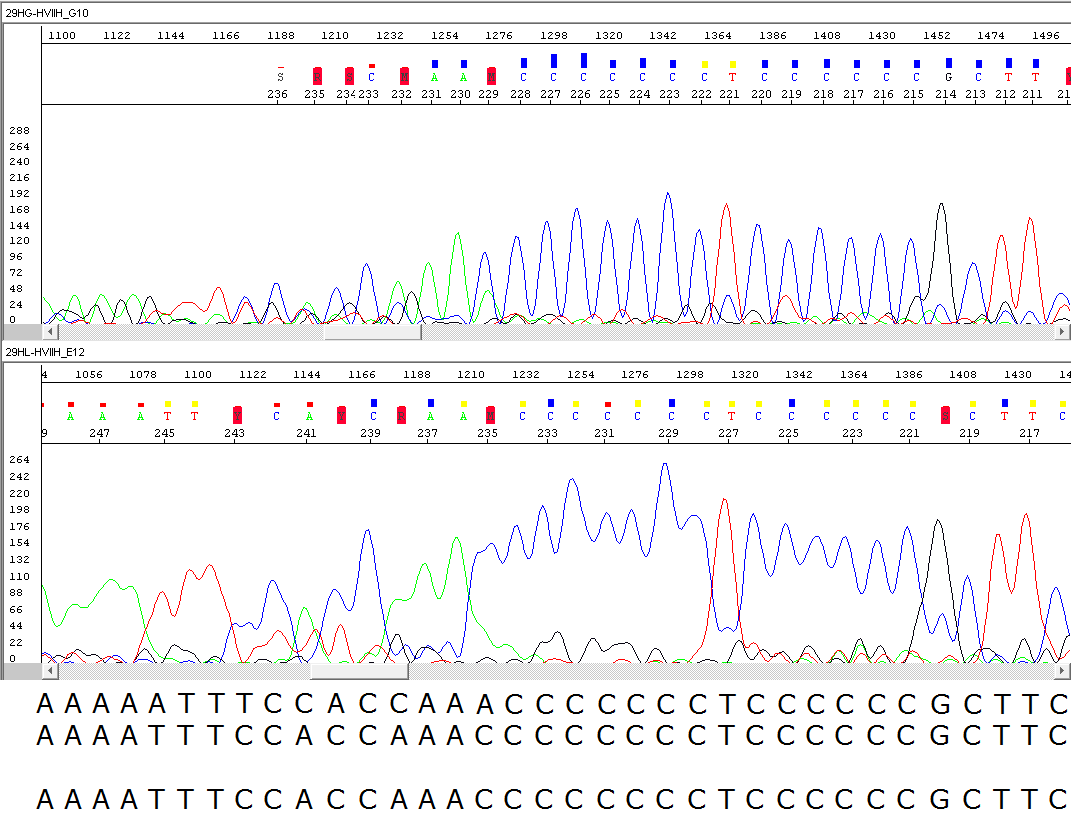 | |
| 29HG and 29HL: 309+C>rCRS/309+C | |
| 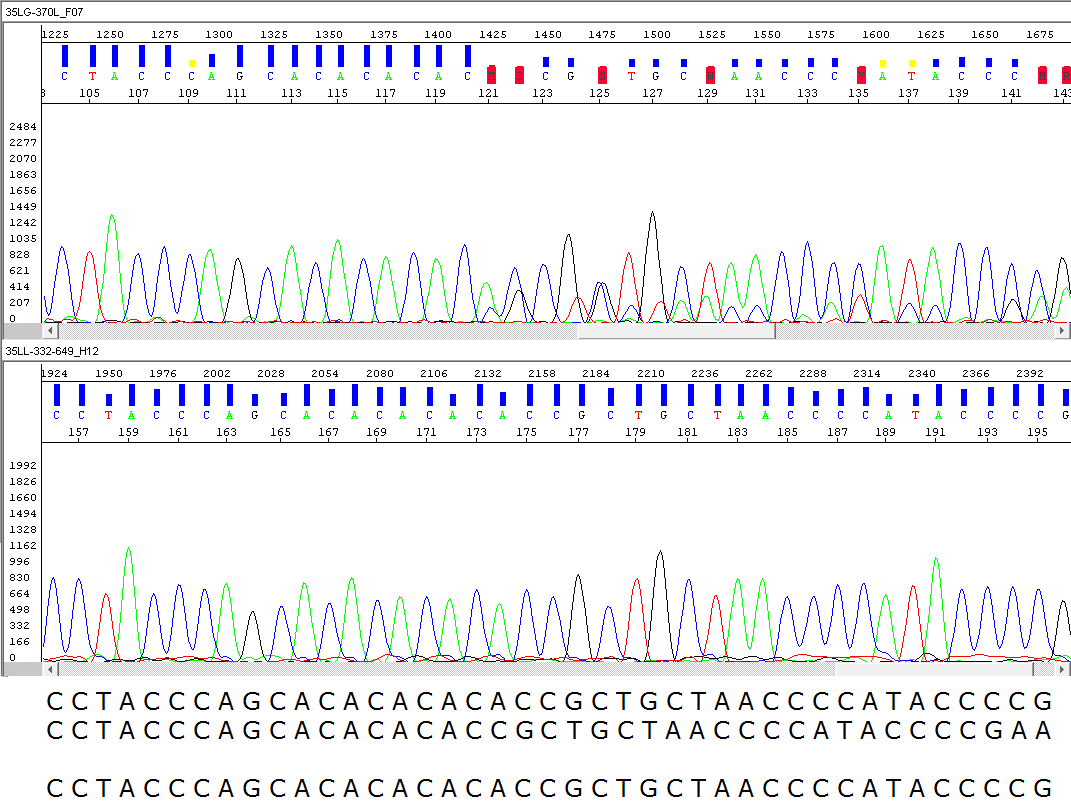 | |
| 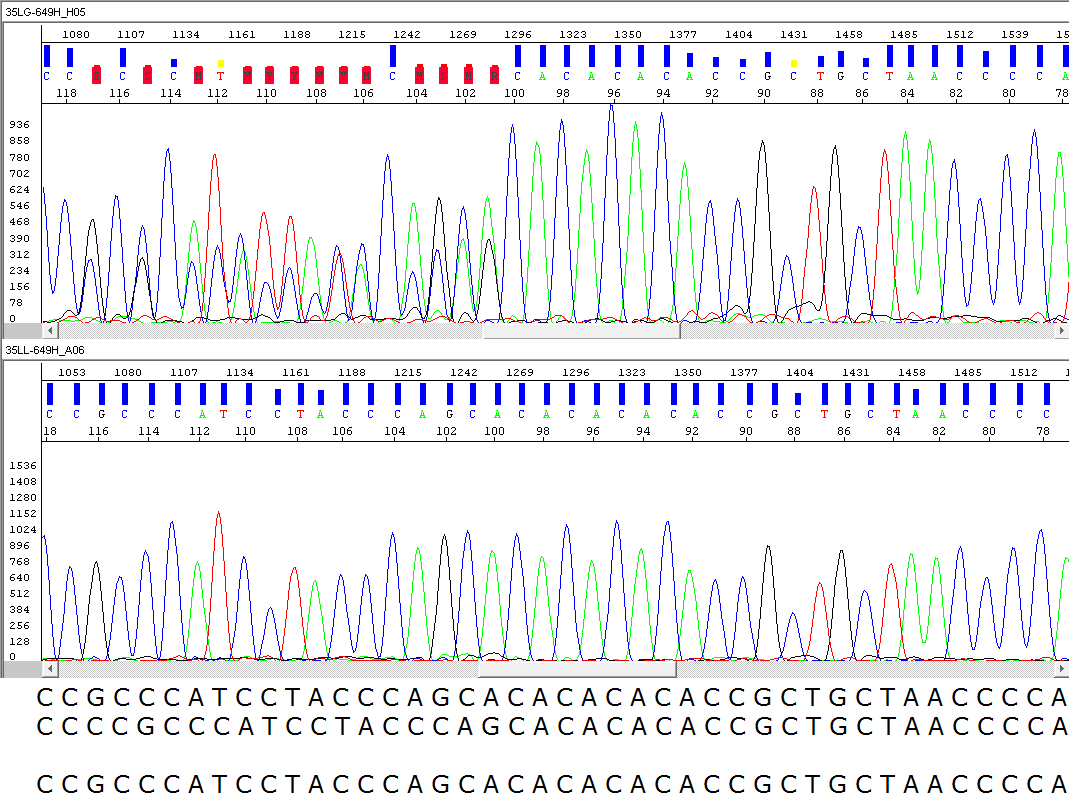 | |
| 35LG and 35LL: rCRS>523-524delAC/rCRS | |
| 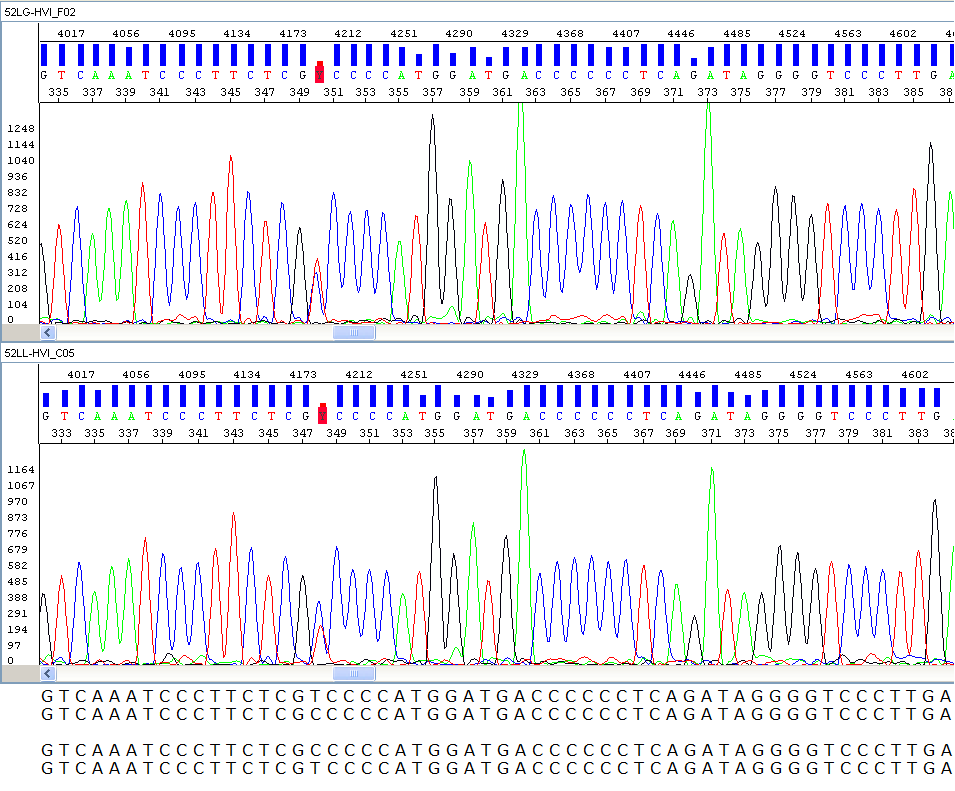 | |
| 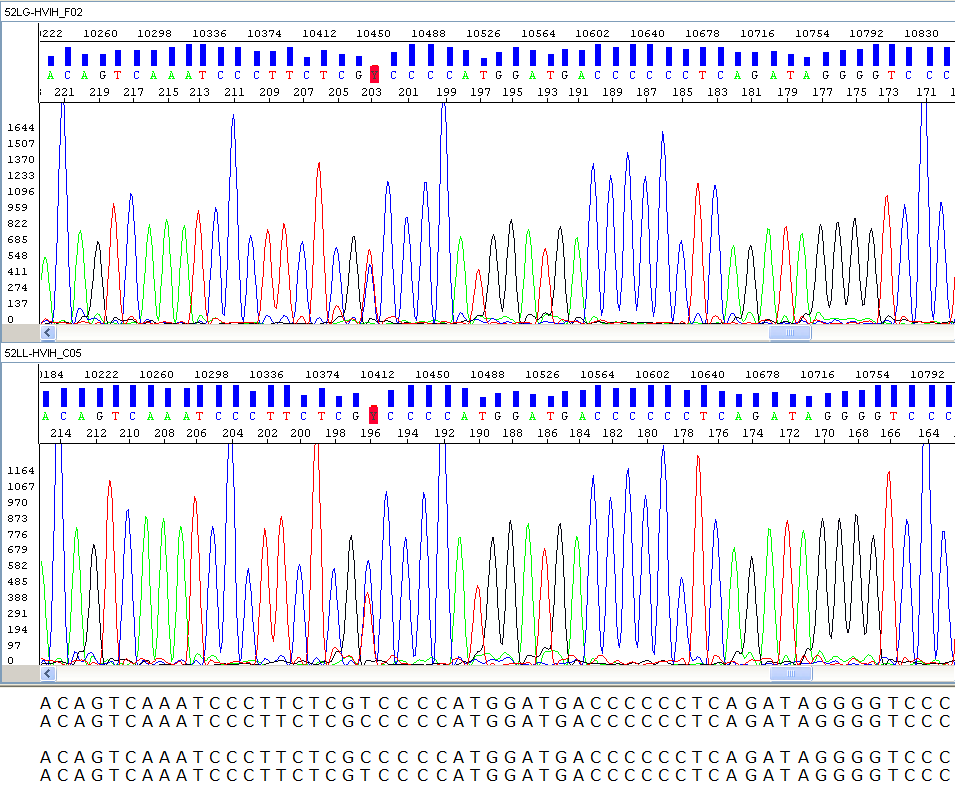 | |
| 52LG and 52LL: 362T>C/362C>T | |
| 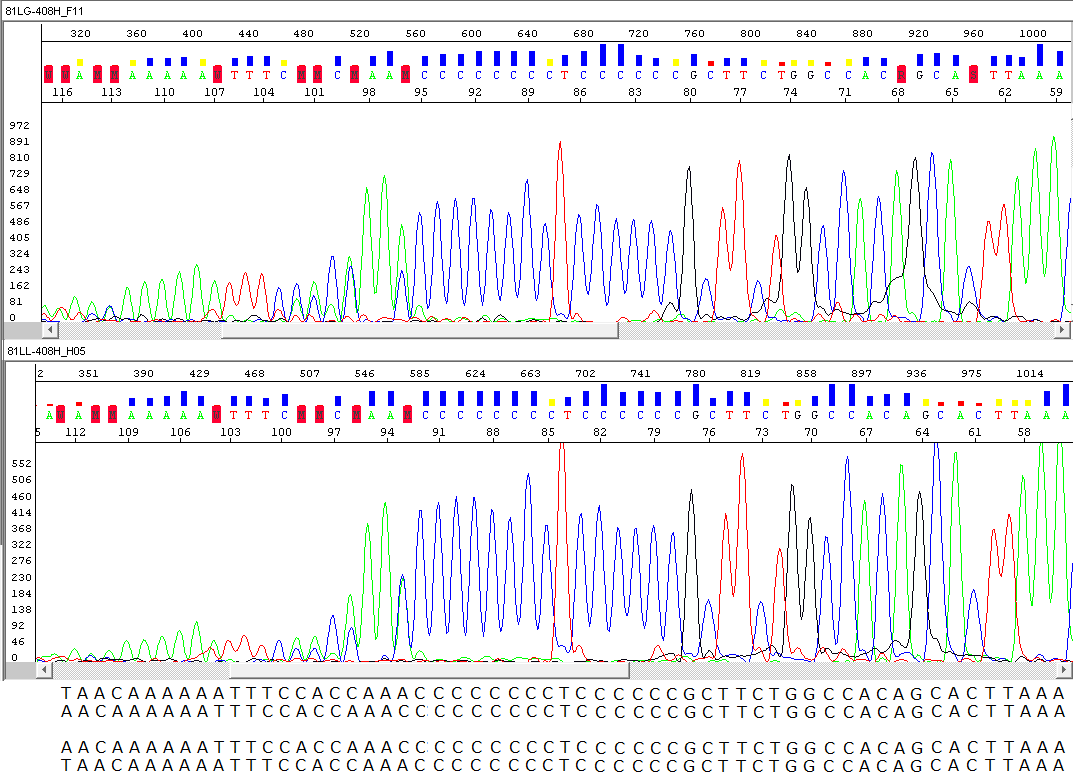 | |
| 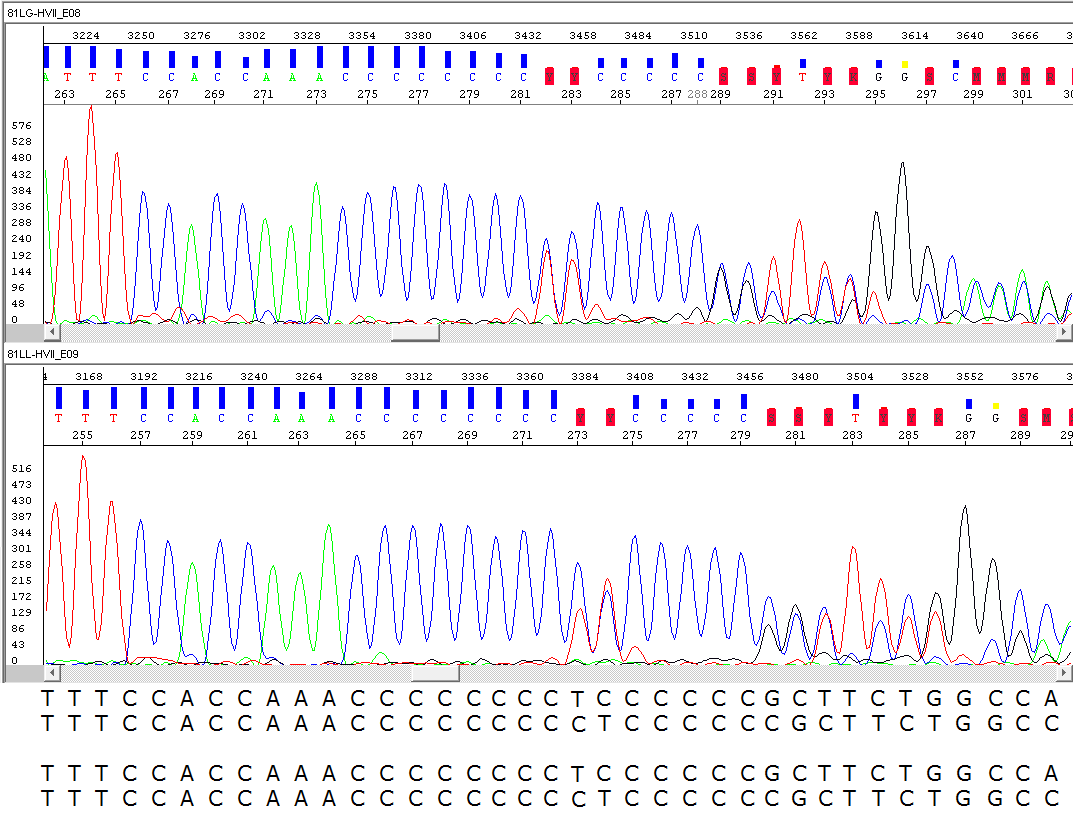 | |
| 81LG and 81LL: 309+C>309+CC/309+CC>309+C | |
| 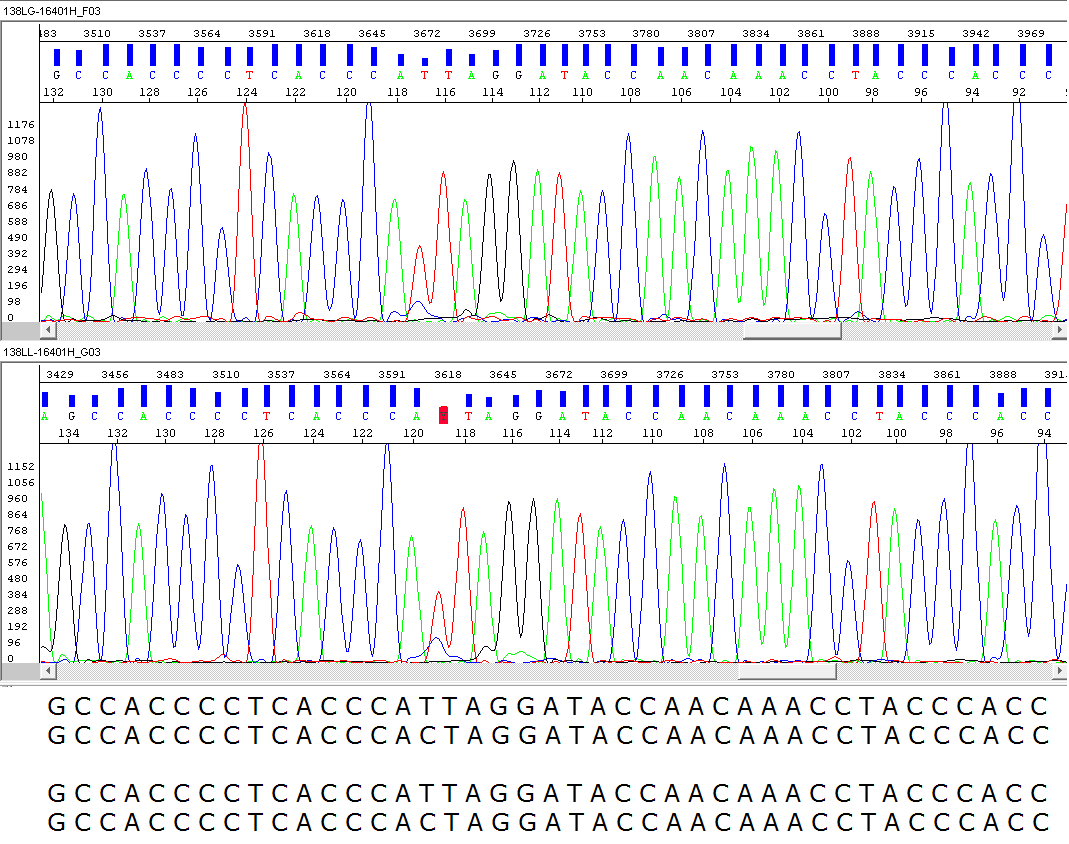 | |
| 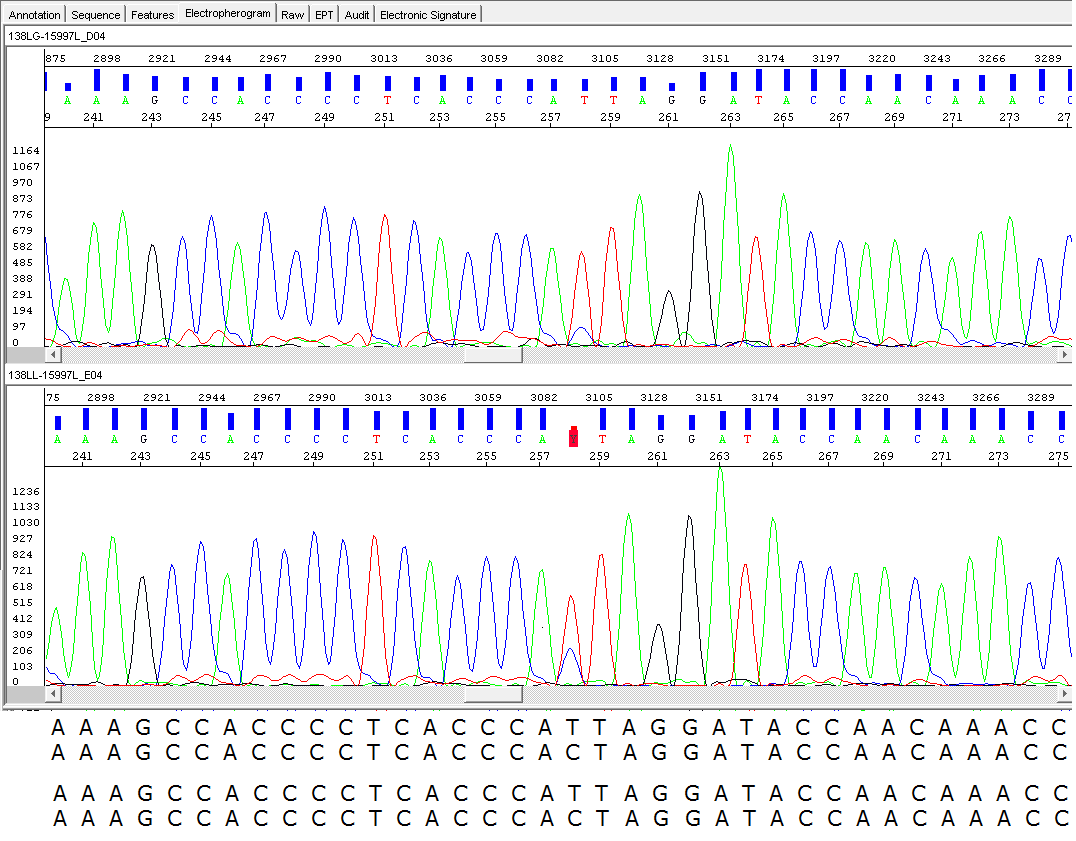 | |
| 138LG and 138LL: 16270T>>C/16270T>C | |
| 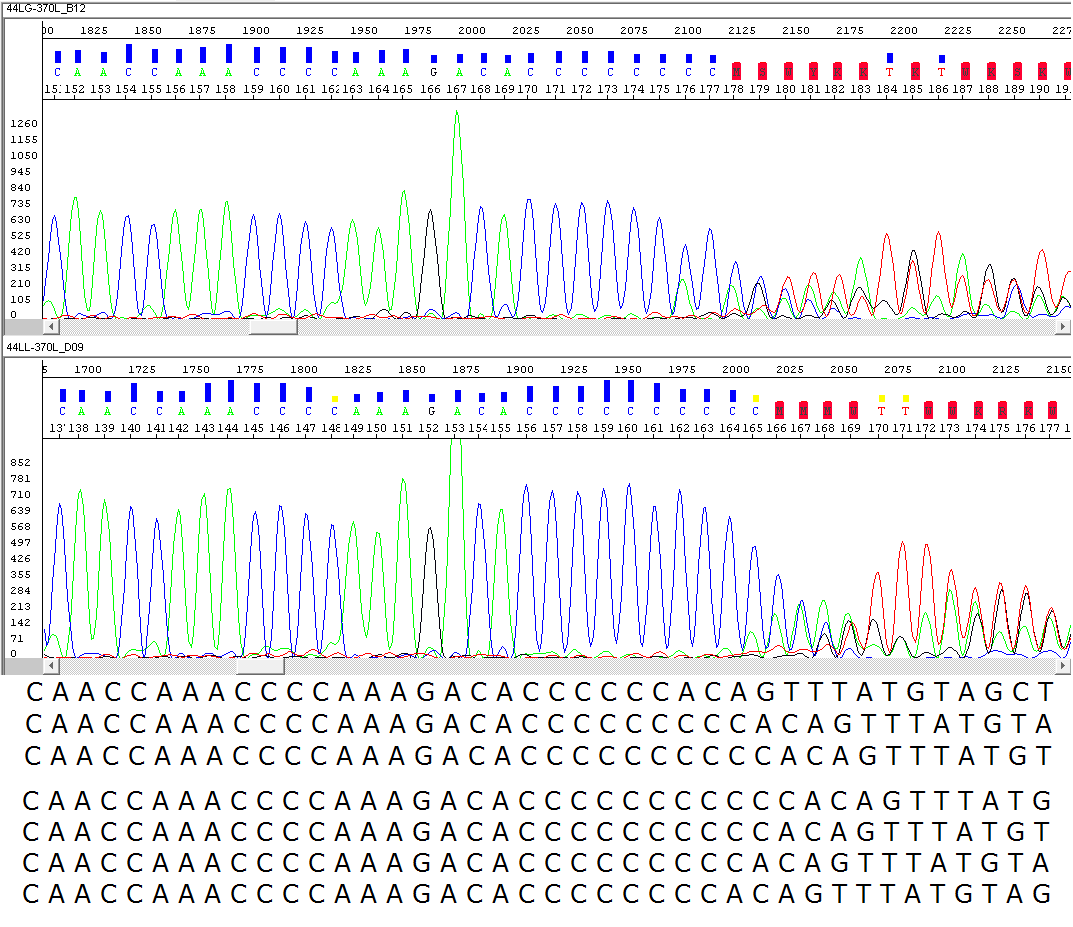 | |
| 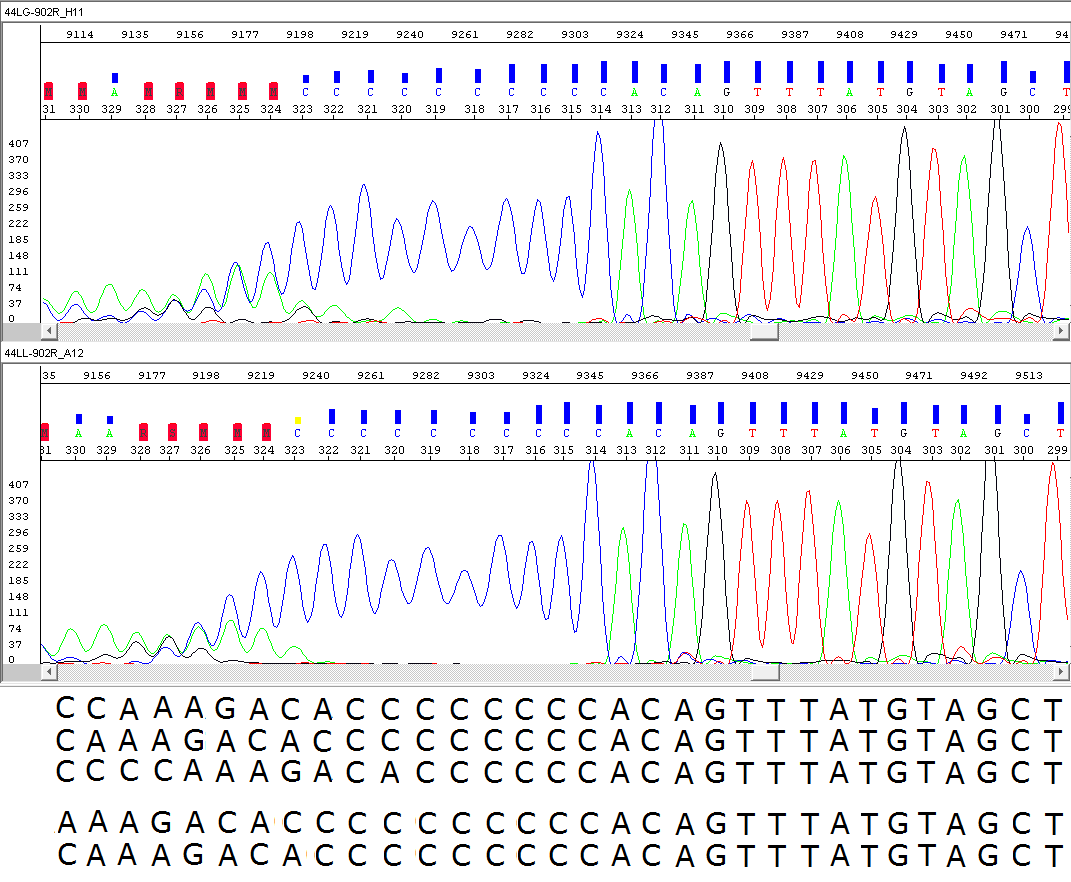 | |
| 44LG and 44LL: 573+2C>rCRS/573+4C>573+3C | |
|  | |
|  | |
|  | |
